# Unlocking the Genetic Diversity and Population Structure of a Wild Gene Source of Wheat, *Aegilops biuncialis Vis.*, and Its Relationship With the Heading Time

**DOI:** 10.3389/fpls.2019.01531

**Published:** 2019-11-22

**Authors:** László Ivanizs, István Monostori, András Farkas, Mária Megyeri, Péter Mikó, Edina Türkösi, Eszter Gaál, Andrea Lenykó-Thegze, Kitti Szőke-Pázsi, Éva Szakács, Éva Darkó, Tibor Kiss, Andrzej Kilian, István Molnár

**Affiliations:** ^1^Agricultural Institute, Centre for Agricultural Research, Martonvásár, Hungary; ^2^University of Canberra, Diversity Array Technologies, Canberra, ACT, Australia; ^3^Institute of Experimental Botany, Center of the Region Haná for Biotechnological and Agricultural Research, Olomouc, Czechia

**Keywords:** *Aegilops biuncialis*, genetic diversity, DArTseq markers, population structure, hierarchical clustering, heading time

## Abstract

Understanding the genetic diversity of *Aegilops biuncialis*, a valuable source of agronomical useful genes, may significantly facilitate the introgression breeding of wheat. The genetic diversity and population structure of 86 *Ae. biuncialis* genotypes were investigated by 32700 DArT markers with the simultaneous application of three statistical methods— neighbor-joining clustering, Principal Coordinate Analysis, and the Bayesian approach to classification. The collection of *Ae. biuncialis* accessions was divided into five groups that correlated well with their eco-geographic habitat: A (North Africa), B (mainly from Balkans), C (Kosovo and Near East), D (Turkey, Crimea, and Peloponnese), and E (Azerbaijan and the Levant region). The diversity between the *Ae. biuncialis* accessions for a phenological trait (heading time), which is of decisive importance in the adaptation of plants to different eco-geographical environments, was studied over 3 years. A comparison of the intraspecific variation in the heading time trait by means of analysis of variance and principal component analysis revealed four phenotypic categories showing association with the genetic structure and geographic distribution, except for minor differences. The detailed exploration of genetic and phenologic divergence provides an insight into the adaptation capacity of *Ae. biuncialis*, identifying promising genotypes that could be utilized for wheat improvement.

## Introduction

The genome of wild relatives of common wheat (*Triticum aestivum* L.) can be considered as a potential reservoir of gene variants for wheat improvement (Schneider et al., 2008; Zhang et al., 2015; Kishii, 2019). Interspecific hybridization is a promising approach to enlarge the genetic diversity of cultivated bread wheat by the chromosome-mediated transfer of the wild alleles present in related species (Jauhar, 1993; Jauhar and Chibbar, 1999; Kishii, 2019). Goatgrasses (*Aegilops*), which comprise 11 diploid, 10 tetraploid, and 2 hexaploid species, are the closest relatives of *Triticum* (van Slageren, 1994). Seven different genomes (D, S, U, C, N, M, and T) were identified in the diploid species, indicating the extreme genetic diversity of the genus. Accessions of several *Aegilops* species are highly resistant to important cereal diseases (Friebe et al., 1996; Marais et al., 2006; Bansal et al., 2017; Olivera et al., 2018), while others shows good tolerance of abiotic stresses such as salt, drought, frost, and heat stress (Rekika et al., 1997; Zaharieva et al., 2001a; Zaharieva et al., 2001b; Molnár et al., 2004; Colmer et al., 2006; Dulai et al., 2014). Some alleles associated with these agronomic traits have already been introgressed from *Aegilops* into the wheat gene pool by the development of wheat-*Aegilops* hybrids and addition or translocation lines (Schneider et al., 2008; Marais et al., 2009; Liu et al., 2011a; Liu et al., 2011b; Olson et al., 2013). However, the genetic potential of *Aegilops* is still largely underutilized. In the case of biotic stresses, the 41 resistance genes that have so far been integrated into the wheat genome originated from only 30 accessions from 12 *Aegilops* species, most of them belonging to the primary gene pool of hexaploid wheat (Zhang et al., 2015). There are numerous *Aegilops* accessions in gene banks in various parts of the world (Monneveux et al., 2000) that have not yet been utilized for wheat improvement, so their introduction into breeding programs would be desirable.

The annual allotetraploid *Aegilops biuncialis* Vis. (2n = 4x = 28; U^b^U^b^M^b^M^b^) is largely autogamous and belongs to the tertiary gene pool of bread wheat (Friebe et al., 1996). *Ae. biuncialis* is native to Mediterranean and Western Asiatic regions and populations can be found in the Aegean, Turkey, Bulgaria, Cyprus, in the western part of the Fertile Crescent, in Cis- and Transcaucasia, and in the southern parts of Russia and Ukraine (van Slageren, 1994; Kilian et al., 2011). The annual rainfall in these habitats ranges from 225–1250 mm and some of them are characterized by a dry summer season with high temperature and high irradiance (van Slageren, 1994). The wide eco-geographical distribution suggests the presence of great diversity in the phenological traits of *Ae. biuncialis*. The heading time, as one of the main phenological factors, is crucial for the ecological adaptation of plants to local conditions. Early flowering, as an avoidance mechanism, may have a major role in the adaptation of plants to a Mediterranean climate by allowing them to escape drought (Araus et al., 2004). The adaptation strategy of wild emmer wheat populations to natural habitats that are characterized by frequent high temperatures in spring includes early flowering (Peleg et al., 2005), while adaptation to habitats with high altitudes involves late heading. Nevertheless, no data have been published on the intraspecific variation of heading time in accessions representing the broad ecological adaptability of *Ae. biuncialis*, which exhibits great genetic diversity.

The genetic diversity of wild species that are gene sources for cultivated wheat is a critical component of research in evolution, population genetics, conservation and breeding (Tahernezhad et al., 2010; Wang et al., 2013; Arora et al., 2017; Edet et al., 2018; Etminan et al., 2019; Singh et al., 2019). Little has been reported about the genetic diversity of *Ae. biuncialis*. Studies based on amplified fragment length polymorphism (AFLP), sequence-specific amplified polymorphism, random amplified polymorphic DNA, and inter-simple sequence repeat molecular markers were used to reveal the genetic variability within the genome of *Ae. biuncialis* accessions (Okuno et al., 1998; Monte et al., 2001; Nagy et al., 2006; Thomas and Bebeli, 2010), but Nagy et al. (2006) concluded that the marker data of only 10 accessions cannot demonstrate the existing genetic variability in *Ae. biuncialis*, so it is essential to investigate a larger diverse collection of genotypes with high marker density for the comprehensive study of genetic diversity. The 5–10 accessions so far assessed using a larger number of loci all originated from narrow geographical regions (Greece, the Iberian Peninsula or Transcaucasia), so do not represent the wide distribution of the species and do not allow an objective picture to be obtained of the genetic diversity existing within *Ae. biuncialis* (Okuno et al., 1998; Monte et al., 2001; Thomas and Bebeli, 2010). Moreover, different parts of the genome may have undergone different evolutionary changes (Kellogg et al., 1996). In the Triticeae, structural rearrangements frequently occur in the pericentric region of the chromosomes after polyploidization (Qi et al., 2006), as also revealed in *Ae. biuncialis* accessions (Molnár et al., 2011). Additionally, the frequency of recombination and the rate of interstitial deletions and insertions of gene loci is much higher in the distal third of the chromosomes than in the proximal two-thirds (Dvorak and Akhunov, 2005). As a consequence, only part of the genetic polymorphisms present in the species can be detected if the genome is not sufficiently covered by molecular markers.

Diversity Array Technology (DArT) was originally developed as a hybridization-based microarray platform to detect polymorphism at the recognition sites of methylation-sensitive restriction enzymes (Jaccoud et al., 2001; Wenzl et al., 2004). More recently, a combination of this genome complexity reduction approach of DArT technology with next-generation sequencing technologies resulted in a sequence-independent, low-cost, high-throughput genotyping by sequencing platform allowing the simultaneous detection of several thousands of polymorphic loci spread over the genome (Wenzl et al., 2004; Tinker et al., 2009; Sansaloni et al., 2011; Kilian et al., 2012). The advanced DArTseq technology has been efficiently utilized for genotyping, genetic diversity analysis, genome-wide association studies and linkage mapping in cultivated and wild relatives of wheat such as Triticale (Badea et al., 2011), rye (Bolibok-Brągoszewska et al., 2014; Gawroński et al., 2016), barley (Comadran et al., 2011), Tibetan wild barley (Cai et al., 2013), durum wheat (Baloch et al., 2017), *Aegilops tauschii* (Kumar et al., 2015), *Triticum monococcum* (Jing et al., 2009), and hexaploid wheat cultivars (Nielsen et al., 2014; Monostori et al., 2017).

In the present work, genetic diversity and its association with the phenotypic variation in heading time were studied in a collection of 86 *Ae. biuncialis* genotypes to obtain a better understanding of the background of its genotypic and phenotypic diversification. To achieve this goal, the genetic relationships between the accessions were analyzed using three statistical methods after the DArTseq genotyping of the plants. Furthermore, the phenological variation pattern was compared with the genetic structure and eco-geographical distribution to obtain information on possible correlations between them. The comprehensive study of how changes in phenological traits relate with genetic diversity will facilitate the utilization of the enhanced adaptability of *Ae. biuncialis*, especially in the light of climate change.

## Materials and Methods

### Plant Material

Eighty-six wild *Ae. biuncialis* Vis. (2n = 4x = 28, U^b^U^b^M^b^M^b^) accessions, collected from 64 sites in 16 countries from Libya to Azerbaijan, were genotyped together with the Mv9kr1 wheat accession on a DArTseq^®^ platform (**Table 1** and **Supplementary Table S1**). This population, originating from a wide range of ecological habitats, is representative of the geographical distribution of the species. The *Aegilops* genotypes were provided by the following germplasm collections: Genebank of the Agricultural Institute, Agrártudományi Kutatóközpont (ATK) (Martonvásár, Hungary), Wheat Genetics Resource Center (Kansas State University, USA), United States Department of Agriculture Agricultural Research Service (Beltsville, MD, USA), and Institute of Plant Genetics and Crop Plant Research (Gatersleben, Germany). The *A. biuncialis* accessions were maintained and grown in the Department of Plant Genetic Resources of ATK Mezőgazdasági Intézet. To avoid hybridization and intercrossing between the genotypes, the isolated accessions were multiplicated by self-fertilization.

**Table 1:** Geographic distribution of the *A. biuncialis* collection maintained in Martonvásár.

Country	No. of accessions	Ratio (%)	Climate zone
Libya	4	4.65	Semi-arid Mediterranean
Bosnia and Herzegovina	4	4.65	Mediterranean and Continental
Serbia	2	2.33	Humid Continental
Macedonia	2	2.33	Humid Continental
Bulgaria	1	1.16	Humid Continental
Kosovo	1	1.16	Mediterranean and Continental
Greece	9	10.46	Mediterranean Humid Continental
Turkey	25	29.07	Mediterranean
Ukraine (Crimean)	3	3.49	Mediterranean and Continental
Cyprus	2	2.33	Mediterranean
Azerbaijan	7	8.14	Semi-arid Mediterranean Humid Continental
Iraq	1	1.16	Semi-arid
France	1	1.16	Mediterranean
Syria	11	12.79	Semi-arid Mediterranean
Jordan	6	6.98	Semi-arid Mediterranean
Israel	1	1.16	Mediterranean
Unknown	6	6.98	
**Total**	**86**	**100**	

The geographic origin of the Ae. biuncialis genotypes can be found in the Plant Inventory Books: USDA ARS, European Wheat Database and Genesys Accession browser. Climatic conditions at the site of origin of the Ae. biuncialis accessions, expressed according to the Köppen-Geiger classification system.

### Field Experiments

The collection of 86 *Ae. biuncialis* accessions was grown under field conditions in three consecutive seasons (2015–16, 2016–17, and 2017–18). The seeds were planted in fall in fields belonging to the Agricultural Institute of ATK (Breeders nursery, Martonvásár, Hungary, geographic coordinates: 47°19’39”N, 18°47’01”E). The soil type at each location was chernozem. Each genotype was grown in a 6 m^2^ plot with 6 × 3 m rows, 50 seeds per row, and a row distance of 0.15 m, as previously described by Mikó et al. (2014). The heading time of each plot was recorded and defined as the period elapsing between January 1 and the day when 50% of the inflorescences reached the DEV59 developmental stage (Tottman, 1987).

Additionally, to monitor the stay-green ability of the plants, the leaf chlorophyll content of each *Ae. biuncialis* genotype was determined twice (on May 12 and 31, 2018) during the grain-filling period using a Soil Plant Analysis Development 502 cholorophyll meter (Minolta Camera Co., Ltd, Tokyo, Japan). At least 10 leaves were measured from randomly selected plants for each genotype on each occasion. For each leaf, the average of three SPAD readings around the midpoints of the flag leaf was taken.

### DNA Extraction and Genotyping

Genomic DNA was extracted from young leaves of 86 *Ae. biuncialis* genotypes together with hexaploid wheat (*Triticum aestivum* L.) genotype Mv9kr1 using Quick Gene-Mini80 (FujiFilm, Japan) with a QuickGene DNA tissue kit (FujiFilm, Japan) according to the manufacturer’s instructions (Cseh et al., 2011).

The DNA of the *Ae. biuncialis* accessions and the Mv9kr1 wheat genotype was sent for a high-throughput genotyping commercial service to Diversity Arrays Technologies Pty. Ltd., Australia (www.diversityarrays.com). The “wheat DArTseq™ 1.0” genotyping by sequencing service was optimized for wheat. This method used the combination of complexity reduction methods developed initially for array-based DArT and multiplex sequencing in Illumina HiSeq2500 instrument (Sansaloni et al., 2011; Kilian et al., 2012). Briefly, DArT markers are DNA fragments obtained by the genome complexity reduction method contains the digestion of genomic DNA by *Pst*I/*Taq*I endonucleases, ligation of the genomic fragments to a *Pst*I adapter, amplification using a primer complementer with the adaptor sequence and transformation into *Escherichia coli*. DNA fragments obtained by this genome complexity reduction method were sequenced by Illumina HiSeq2500.

The markers developed by the above mentioned “wheat DArTseq™ 1.0” genotyping by sequencing platform have been included in **Supplementary Data S1**. The obtained silicoDArT marker set was filtered on the basis of individual marker-related statistics. After removing markers with inappropriate quality control parameters, including call rate <90%, reproducibility <95% and minor allele frequency < 2%, the 0/1 binary matrix of the remaining 32,700 markers was used in the subsequent analysis.

### Analysis of Population Structure and Phylogenetic Relationship

In order to assess the population structure of the *Ae. biuncalis* accessions, three different statistical methods were adopted and compared. First, a clustering approach based on the Bayesian model was applied to estimate the real number of subpopulations (K) using the admixture model of STRUCTURE software 2.3.4 with correlated allele frequencies (Pritchard et al., 2000). Three independent runs were performed for each hypothetical number of subpopulations (K) from one to eight applying a burn-in period of 100,000 iterations followed by 100,000 Markov Chain Monte Carlo iterations to obtain a precise parameter estimate. The most probable number of subpopulations was determined by means of the ΔK method using STRUCTURE HARVESTER software (Evanno et al., 2005). Each genotype was assigned to one subpopulation based on its membership probability. Principal coordinate analysis was then carried out using PAST software version 3.12 to visualize the genetic stratification within the *Ae. biuncialis* collection based on genetic correlations among individuals (Hammer et al., 2001). Thirdly, the phylogenetic relationship between the 86 accessions was estimated using PAST software with the neighbor-joining method based on marker data. The genetic distance matrix based on Jaccard’s similarity coefficient was applied to construct a phylogenetic tree. The neighbor-joining dendrogram was generated using bootstrap analysis with 1,000 replicates. The wheat genotype Mv9kr1 was used as outgroup species in order to define the root position of the phylogenetic tree.

### Statistical Analysis

The phenotypic values of the *Ae. biuncialis* accessions were grouped based on the subpopulations obtained in the STRUCTURE cluster analysis. The variations in heading time among the subpopulations were analyzed for 3 years using descriptive statistics: mean, standard deviation, boxplot, and histogram (Excel 2016, Microsoft Company).

One-way analysis of variance (ANOVA) (SPSS 16.0, IBM) was conducted to compare the heading time of the *Ae. biuncialis* subpopulations in each year at the P < 0.05 significance level. In addition, the significant differences in the heading time of each subpopulation were examined among the 3 years by means of one-way ANOVA (SPSS 16.0). Bivariate correlation of the heading times of the *Ae. biuncialis* accessions among the 3 years was analyzed pair-wise by means of Pearson coefficient (SPSS 16.0). Linear regression analysis was carried out for each subpopulation among the years.

The principal component analysis of the Statistica 6 software was used to study associations between the heading times of the *Ae. biuncialis* accessions by visualizing the grouping pattern of phenotypic variations.

The stay-green trait (delayed foliar senescence) was determined by calculating differences between the SPAD values measured on the two dates. Pearson and rank correlation (Spearman, Kendall tau) coefficients were applied to analyze the dependence and relationship between the heading time and the differences in SPAD values at the P < 0.01 significance level (SPSS 16.0).

## Results

### Population Stratification

**Figure 1:**
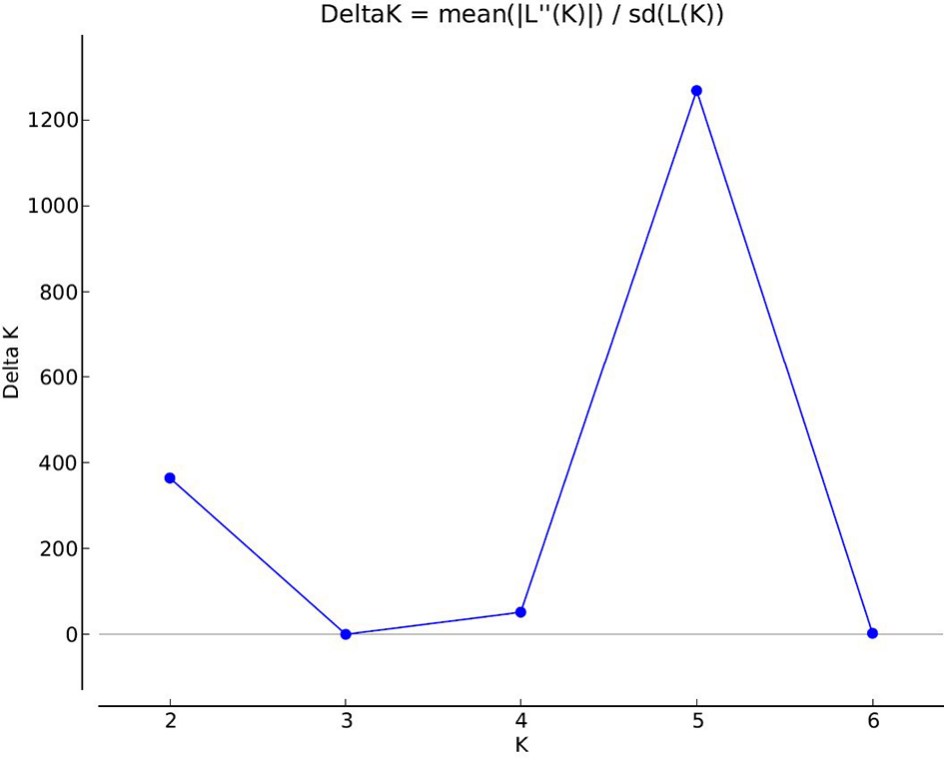
Estimation of the genetically most probable number of *Ae. biuncialis* subpopulations based on Evanno’s Delta K method (Evanno et al., 2005). The maximum value of delta K occurred at K = 5 indicates that the investigated *Ae. biuncialis* collection can be devided into five hypothetical subpopulations.

A total of 47,777 polymorphic dominant silicoDArT markers were generated from 86 *Ae. biuncialis* accessions originating from different eco-geographical habitats (**Supplementary Data S1**). The silicoDArT marker set was filtered for various quality parameters (call rate, reproducibilty, minor allele frequency) and reduced to 32,700 markers. Based on the marker data, the genetic diversity in the *Ae. biuncialis* collection was analyzed using the Bayesian clustering approach performed with STRUCTURE software. After the STRUCTURE analysis had been run for K = 1 to K = 8, the most likely number of subpopulations was estimated with the STRUCTURE Harvester software following the ΔK method. The maximum ΔK value occurred at K = 5 (**Figure 1** and **Supplementary Figure S1**). Each genotype was assigned to one of the subpopulations based on a membership probability coefficient > 0.51.

**Figure 2:**
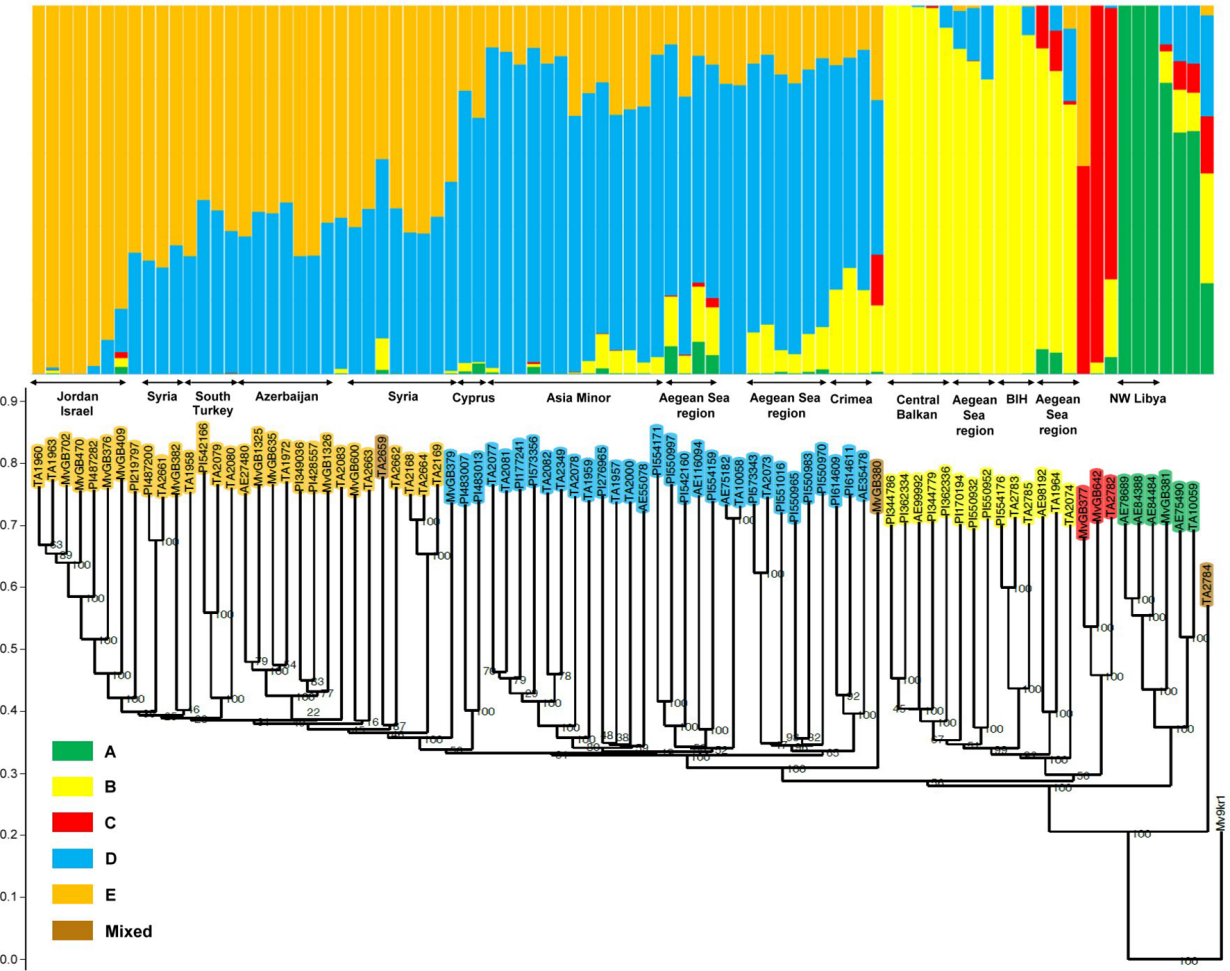
Comparison of grouping patterns based on STRUCTURE analysis at K = 5 and the neighbor-joining dendrogram in a collection of 86 *Ae. biuncialis* accessions. The order of the genotypes on the STRUCTURE plot matches that represented on the phylogenetic tree. The wheat genotype Mv9kr1 is represented as an outgroup accession in the dendrogram. Genotypes belonging to the same clade represent similar membership probability in the relevant subpopulation and are from a common area within the larger geographic regions. BIH: Bosnia and Herzegovina.

**Figure 3:**
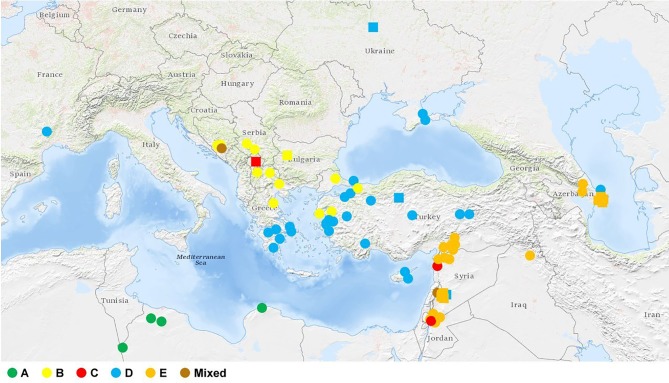
Geographic origin of the *Ae. biuncialis* collection. Circles represent accessions with known geographic location. Where only the country of origin is known, it is indicated by a square representing the capital. There is no information about the origin of six accessions in the collection. The different colors of the symbols (circle, square) in the figure represent the clusters (or subpopulations) corresponding to the population structure obtained by STRUCTURE analysis.

The *Ae. biuncialis* accessions were grouped into five clusters A, B, C, D, and E with 6, 14, 3, 31, and 29 accessions, respectively. Three accessions had mixed allelic patterns that could not be assigned to any of the subpopulations with a probability of more than 50% (mixed group). The accessions clustered in accordance with their origin: the accessions in cluster A originated mainly from North Africa; cluster B represented genotypes from the Central Balkans, North Greece, and West Turkey; the three genotypes in cluster C originated from different geographic regions (Balkans and Near East); cluster D contained accessions from Asia Minor, the Crimean Peninsula, and Southern Greece (Peloponnese); while cluster E comprised genotypes from the Levant region (Jordan, Syria, and South Turkey) and Azerbaijan (**Figures 2** and **3**).

Based on the membership probability of genotypes, subpopulations A, B, and C could be clearly separated, whereas D and E were less distinct due to their similar share in the genome of several accessions (**Figure 2**). Subpopulation A was differentiated and sub-divided according to the membership probability of each cluster: Three of the six accessions (AE78689, AE84388, and AE84484) segregated into a homogeneous subgroup, but the other three formed a heterogeneous subgroup. Subpopulation A appeared in different proportions in the genome of the two subgroups. Subpopulation D contributed to the gene pool of some accessions in subpopulation B whereas the B subpopulation was also detectable in several genotypes in subpopulation D. These accessions within the two subpopulations occupy a common eco-geographic area in Greece and West Turkey (Aegean Sea region), indicating admixture events between subpopulations B and D. In subpopulation C, other subpopulations also significantly contributed to the genome of two accessions (TA2782 and MvGB377). Subpopulation B was revealed in genotype TA2782 from Kosovo, whereas subpopulation E was detected in the genome of MvGB377, originating from Jordan. The third, Syrian genotype (MvGB642) had a 97% membership probability in cluster C.

### Analysis of Genetic Diversity

To refine the genetic relationship between the *Ae. biuncialis* accessions, the genetic similarity within the population was calculated based on Jaccard’s coefficient. The average genetic similarity between all 86 genotypes was estimated to be 0.45 and ranged from 0.28 to 0.96, indicating that the *Ae. biuncialis* population had large genetic variability (data of the genetic similarity matrix not shown). The phylogenetic dendrogram split the *Ae. biuncialis* collection into five separate branches, which had a good fit with the subpopulations (A-E) revealed by STRUCTURE analysis (**Figure 2**). The pair-wise genetic similarity of the accessions was studied within each subpopulation, of which cluster D had the lowest average value (0.51) and the widest range (0.41–0.96), indicating its broad genetic diversity. The *Ae. biuncialis* accessions were genotyped together with wheat accession, Mv9kr1 which was used as an outgroup accession for constructing the phylogenetic dendrogram for the *Ae. biuncialis* collection.

Based on the STRUCTURE and neighbor-joining analyses results, the subpopulations could be further divided into subgroups (or intra-cluster lineage), where genotypes from common areas share similar genetic ancestry and show high genetic similarity (**Figure 2**). This intra-cluster differentiation was in agreement with the provenance of the genotypes within the larger geographic regions. Cluster B could be separated into subgroups originating mainly from Bosnia and Herzegovina, the central area of the Balkans and coast of the Aegean Sea (North Greece and Northwest Turkey). Subpopulation D was divided into clades springing from Asia Minor, Crimea and region of the Aegean See (South Greece and West Turkey). Based on their genetic similarity, three accessions from Cyprus and Syria (MvGB379, PI483007, and PI483013) could be discriminated clearly in subpopulation D as showing a close genetic relationship with subpopulation E. The latter could be partitioned into distinct subgroups representing their geographic origin, such as Jordan, Azerbaijan, Syria and the southern part of Turkey.

**Figure 4:**
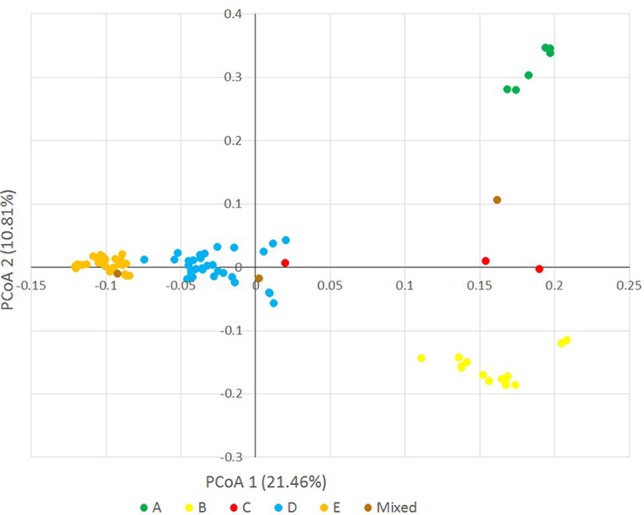
Principal coordinate analysis on the *Ae. biuncialis* accessions based on 32,700 DArT markers. Different colors indicate different subpopulations (A–E) in the population.

Principal coordinate analysis was used as an alternative way of analyzing and visualizing the population structure. The first three principal coordinates explained 38.428% of the genotypic variance (PCo1: 21.46%, PCo2: 10.81%, PCo3: 6.15%), and also discriminated the accessions of clusters A, B, D and E, confirming the STRUCTURE result (**Figure 4**, **Supplementary Figures S2** and **S3**).

### Phenotypic Evaluation of Heading Time

The heading time, as a phenological trait, was grouped according to the subpopulations obtained by cluster analysis (**Supplementary Table S2**). One-way ANOVA was carried out in order to study the differences between the heading time of the five subpopulations within each year, and to analyse the differences in each subpopulation among the 3 years. Although subpopulations A and C had much smaller sample size than the others, they were not excluded from the statistical analysis.

**Figure 5:**
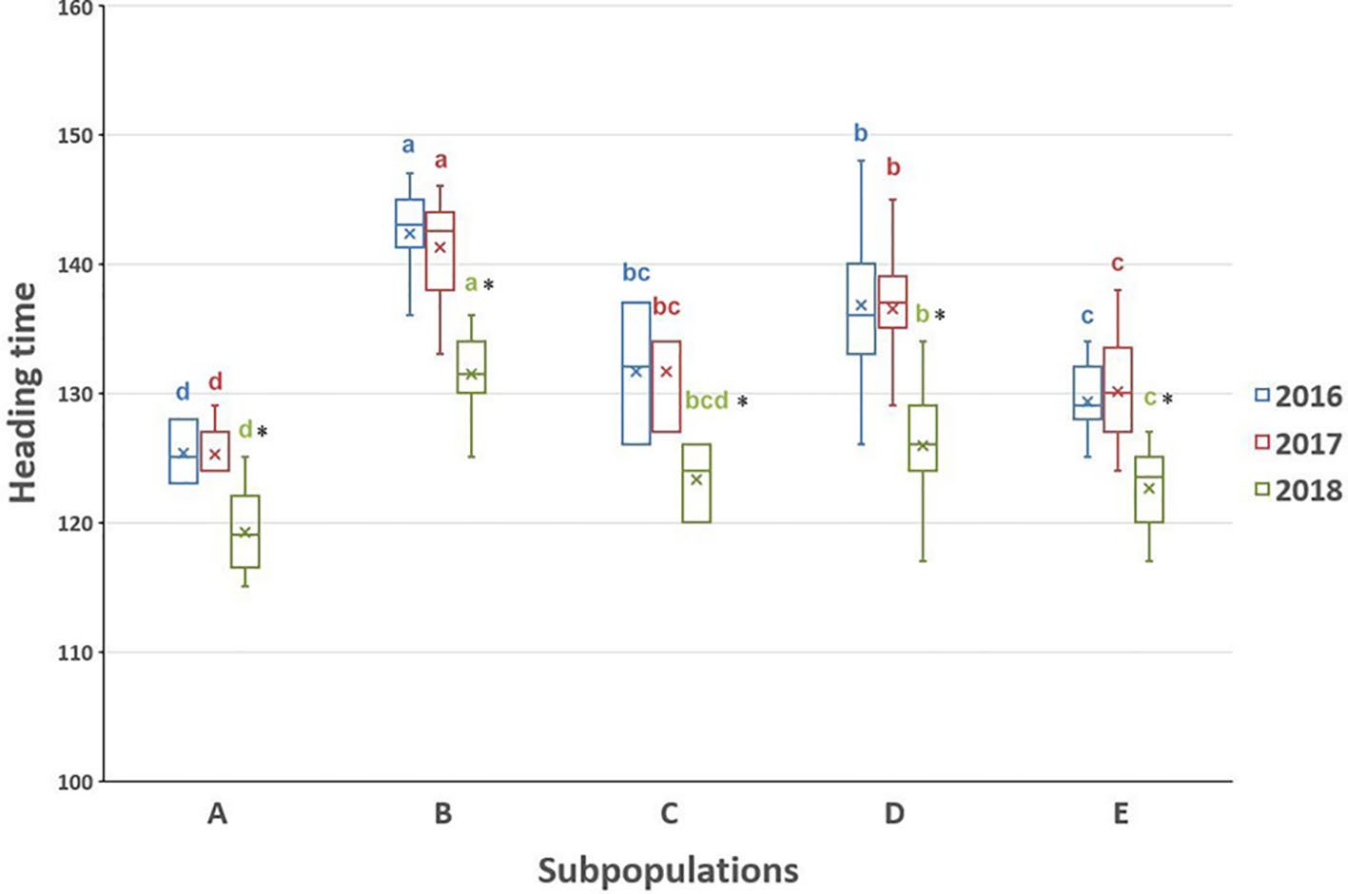
Boxplot chart of the heading times of the *Ae. biuncialis* subpopulations **(A–E)** showing the mean, median and range of the phenotypic data in 3 years. The heading time trait was expressed in number of days elapsed from January 1 to the DEV59 developmental stage. Different letters indicate significant differences between the subpopulations within the same year at P < 0.05, using one-way analysis of variance. *Indicates the heading time in 2018 differs significantly from the others within relevant subpopulation at the P < 0.05 level.

**Figure 6:**
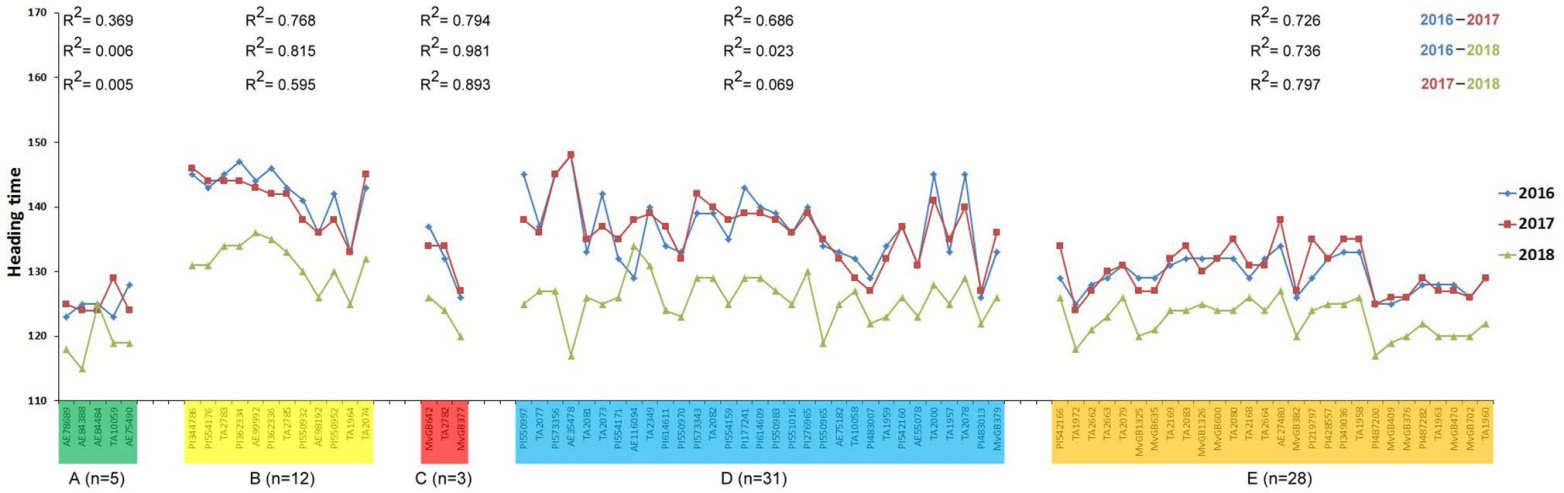
Correlation of the heading times of *Ae. biuncialis* accessions among three different seasons. Heading time was defined as the number of days required to reach the DEV59 developmental stage from January 1. Color codes indicate different subpopulations **(A–E)** identified from STRUCTURE analysis. Number of accessions (n), clustered into one subpopulation, are represented in the figure. The R^2^ values of pair-wise correlation for each subpopulation among different seasons are included in the figure.

The heading time within each subpopulation differed not significantly among 2016 and 2017, while the heading time values in the 2018 season were considerably shorter than in the other 2 years (**Figure 5** and **Supplementary Figure S4**), which was confirmed by the correlation analysis (**Figure 6**). The difference could be explained by the very different precipitation and temperature conditions during the spring months that are critical for the flowering period (**Supplementary Figure S5**). One-way ANOVA of the heading time within 1 year pointed out significant differences between four subpopulations (A, B, D, and E) in the *Ae. biuncialis* collection (**Figure 5** and **Supplementary Figure S4**). The subpopulation C was not separated from those of subpopulations D and E; and in 2018 the subpopulation C differed not from those of A, D, and E.

**Figure 7:**
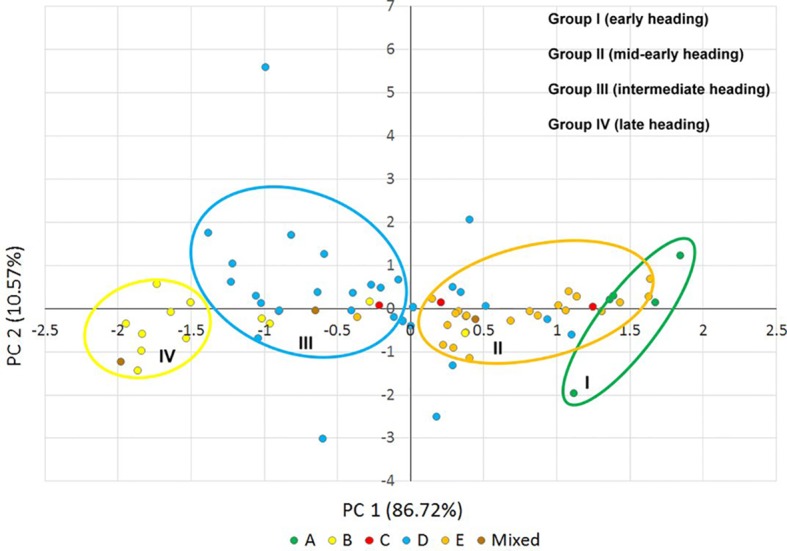
Scatter plot of principal components 1 and 2, explaining 97.29% of the phenotypic variance in the heading time (2016, 2017, and 2018) of the *Ae. biuncialis* collection. Different colored dots indicate the *Ae. biuncialis* accessions in the subpopulations (A–E) obtained from STRUCTURE analysis. Groups I–IV indicate the phenotypic categories for heading time.

To visualize the correlation between genetic diversity and phenotypic variation, principal component analysis was performed for the heading time of the *Ae. biuncialis* accessions (**Figure 7**, **Supplementary Figures S6** and **S7**). The first two components explained 97.29% of the phenotypic variance and grouped the majority of genotypes into four phenotypic categories, which corresponded with the genetic structure and geographic distribution of the population in spite of the fact that they showed not clearly differenctiation (**Figure 7**). Since the third principal component represented only 2.72% of the variation, it was not taken into account in the phenotypic classification. Subpopulations A and E correlated to group I and group II, respectively, exhibiting the earlier heading phenotypes, whereas subpopulation D largely coincided with the intermediate group III. Subpopulation B corresponded the group IV, which had the latest heading date (**Figure 7**). The heading time of subpopulation C could not be classified in a separate phenotypic group, which was in accordance with the results of one-way ANOVA.

### Analysis of Delayed Foliar Senescence

The relationship between delayed foliar senescence (stay-green trait of plants) and the heading time was also investigated. At the first measuring date (May 12), the average SPAD values of the genotypes ranged between 42 and 61, indicating that these plants were still green (**Supplementary Table S3**, **Supplementary Figures S8** and **S9**). In the second phase (May 31) SPAD values could not be recorded for seven *Ae. biuncialis* accessions, because the green color intensity of their leaves was below the detection level due to their advanced maturity. For the other genotypes, a significant correlation was revealed between the heading time and the difference in SPAD values based on the Pearson and rank coefficients. This revealed eight genotypes which had early heading times, but exhibited the small differences in SPAD values that are characteristic of the stay-green phenotype (**Supplementary Table S3** and **Supplementary Figure S10**).

## Discussion

### Genetic Structure of the *Aegilops biuncialis* Collection

The present study is the first report on the use of the DArTseq platform to estimate the intraspecific genetic diversity of *Ae. biuncialis* on accessions representing the natural distribution area of the species. Three statistical methods gave consistent results for the genetic diversity and population structure: The *Ae. biuncialis* accessions clustered into five subpopulations in accordance with their place of origin. Three genotypes (MvGB380, TA2784, and TA2659) could not be classed into any of the distinct subpopulations based on their membership probability and these showed the lowest genetic similarity to the other accessions, indicating that this mixed group was the most distant genetically from the other genotypes in the population.

Based on random amplified polymorphic DNA markers, *Ae. biuncialis* accessions from Transcaucasia were classed by Okuno et al. (1998) in two clusters, which corresponded with groups from the eastern shores of the Black Sea and the western shores of the Caspian Sea, while *Ae. biuncialis* subpopulations from the central and southern parts of the Iberian Penninsula were clearly discriminated by AFLP markers that correlated with their geographical distribution (Monte et al., 2001). Based on simple sequence repeat, AFLP, and single nucleotide polymorphism markers, *Ae. tauschii* genotypes were grouped in two lineages, showed clear differentiation (Pestsova et al., 2000; Mizuno et al., 2010; Jones et al., 2013; Wang et al., 2013; Arora et al., 2017; Singh et al., 2019). Large range of genetic similarity (0.61 to 0.99) was reported for *Ae. tauschii* by Sohail et al. (2012), who used DArT markers to study the genetic relationship between 81 genotypes representing the eco-geographic distribution of the species. In the present study the *Ae. biuncialis* collection had a relatively wide genetic variability (0.28 to 0.96). When comparing the genetic diversity of various *Aegilops* species, Kilian et al. (2007) found lower genetic similarity in *Aegilops speltoides* (0.656) and *Ae. tauschii* (0.75) than in *Aegilops sharonensis* (0.84), *Aegilops searsii* (0.825), and *Aegilops longissima* (0.812). Species in the *Sitopsis* section, except for *Ae. speltoides*, have limited distribution in the Levantine, representing a smaller geographical area in the eastern Mediterranean Basin (van Slageren, 1994; Mendlinger and Zohary, 1995; Millet, 2007; Kilian et al., 2011). The narrow geographic range of *Ae. sharonensis*, restricted to the coastal areas of Levantine, could explain the high average genetic similarity (0.82) estimated between the genotypes (Olivera and Steffenson, 2009; Olivera et al., 2010). AFLP marker analysis on a set of *Aegilops geniculata* genotypes revealed that accessions originating from the main regions of the Mediterranean Basin could be classed into seven groups (Arrigo et al., 2010). Six of the seven groups had a strong bio-geographical structure, derived from the northern parts of the Mediterranean, whereas the remaining one exhibited less genetic structuring, representing the southern Mediterranean areas, including North Africa, Levantine, and the Bosphorus Strait. In contrast, the *Ae. biuncialis* population in the southern and eastern Mediterranean regions could be divided into distinct subpopulations, where subpopulation A occupied North Africa, subpopulations E and C were characteristic of Levantine and subpopulation D was located mainly in Asia Minor. The *Ae. biuncialis* collection, fragmented in the eastern part of the Mediterranean Basin, showed a distinct phylo-geographical pattern, indicating the extremely large genetic diversity of the species.

The subpopulations of the *Ae. biuncialis* collection could be further divided into subgroups, resulting in intra-cluster differentiation, which may explain the admixture events between the genotypes (**Figure 2**). Three of the six accessions in subpopulation A had a close genetic relationship, suggesting that the higher rate of gene flow between them may have resulted in a homogeneous subgroup. They differ phenotypically from the rest of the subpopulation as they have an earlier heading date. As this homogeneous subgroup is derived from the northwest part of Libya, they were presumably isolated from the other members of the subpopulation while adapting to the arid climate. The natural distribution areas of certain accessions, representing an admixture of subpopulations B and D, overlap in the region of the Aegean Sea, suggesting that the genome shared by the two subpopulations was formed due to the intercrossing of parental genotypes derived from the different subpopulations. Three accessions (MvGB379, PI483007, and PI483013) were grouped in subpopulation D based on their membership probability, whereas they also showed a closer phylogenetic relationship with some Syrian genotypes from subpopulation E. As both subpopulations have made similar contributions to the genome of these accessions, their genetically similar germplasm and recent common ancestor suggest that the two clusters have not yet fully separated phylogenetically or that intraspecific hybridization has occurred between them. Subpopulations B and C contributed in similar proportions to the genome of the accession TA2782 originating from Kosovo, postulating an earlier admixture event between ancestral genotypes belonging to different subpopulations. Although three genotypes are not enough to represent the native distributional range of subpopulation C, the TA2782 accession seems to have developed outside this area and may have reached Kosovo as a result of migration. Genotypes AE75182 and TA10058 have close genetic similarity (0.95) in spite of being from distant regions. TA10058 originated from Azerbaijan, but was found well away from the natural distribution area of its subpupolation, so it might be an introduced accession. Herbivores, as vectors, can support the long distance dispersal of plants (Janzen, 1984; Vellend et al., 2003; Römermann et al., 2005), which is enhanced by human activities, such as pastoralism (Blondel, 2006). On the other hand, human-caused climate change may have an impact on the expansion of numerous species (Parmesan and Yohe, 2003; Thomas et al., 2004; Hickling et al., 2006; Jarvis et al., 2008; Devictor et al., 2012). The occurrence of two genotypes (PI614609 and PI614611) in the Crimean Peninsula corresponds to the prognosis that predicts a probable shift in the natural range of *Ae. biuncialis* to the Azov Sea in response to global warming (Ostrowski et al., 2016).

The large genetic diversity of *Ae. biuncialis*, enabling its adaptation to various climatic conditions (Zaharieva et al., 2004; Zaharieva and Monneveux, 2006), may serve as a rich source of potential genes to improve the adaptive capacity of cultivated wheat. It has been reported that some *Ae. biuncialis* accessions have good tolerance of heat and drought stress (Molnár et al., 2004; Dulai et al., 2014) and others are promising for resistance to rust diseases (Molnár-Láng et al., 2014; Olivera et al., 2018).

### Phenotypic Diversity Pattern

Based on one-way ANOVA and correlation analysis (**Figures 5** and **6**), the heading times differed among subpopulations similarly in each year in spite of the fact that the heading times of all subpopulations was shorter in 2018 than in 2016 and 2017. The extrem weather conditions, observed in the 2018 season, can explain the low level of correlation and shorter phenotypic values. Significant differences in heading date was found between four subpopulations (A, B, D, and E), corresponding with genetic relatedness and geographic origin with the exception of subpopulation C, which could not be separated from subpopulations D and E (**Figures 5** and **7**). In subpopulation C, the variation pattern of the heading date suggested that the admixed genomes of these accessions represented a diverse gene pool, and had different geographic origin. However, subpopulation C contained relatively few accessions, so no additional conclusions can be drawn on its phenotypic diversity.

Subpopulations with different phenotype are originating from eco-geographic regions that can be characterized by different agro-climatic conditions. Subpopulation A from North Africa had early phenotype which is prevalent in the semi-arid area, whereas subpopulation B and D from the Balkans and Asia Minor exhibited later heading times, which are predominant in the Continental and Mediterranean regions, respectively. The phenological trait patterns of the subpopulations correlated largely with the climatic conditions found in the relevant geographical areas, suggesting that the heading time diversification might be an adaptive change for *Ae. biuncialis*. The broad genetic diversity and its association with adaptive changes in heading date allowed the species to spread widely. Nevertheless, genealogically-based chloroplast DNA analysis could lead to a more comprehensive identification of the ancestral and derived sublineages and a better understanding of the course of the geographic spread of *Ae. biuncialis*.

The *Aegilops triuncialis* accessions in California, which evolved due to the independent introduction of a few Eurasian genotypes (Peters et al., 1996), were grouped using microsatellite markers into three lineages, each of which had different eco-geographical distribution and showed significant differences in flowering time (Meimberg et al., 2006; Meimberg et al., 2010). However, the phenotypic diversity pattern of the invasive lineages cannot express the intraspecific variation for flowering time because the introduced accessions represent only a part of the natural distribution area of *Ae. triuncialis*.

The correlation between the population structure and flowering time diversification in a collection of 200 Ae. *tauschii* accessions was studied by Matsuoka et al. (2008) using chloroplast DNA geneological analysis. Two of the four major haplogroups (HG7 and HG16) were phenotypically heterogeneous, representing a large proportion (83.5%) of the population. When comparing the DArT marker-based genetic divergence and phenological variation pattern of *Ae. tauschii*, Matsuoka et al. (2015) found that the intraspecific lineage structure was associated with changes in the flowering trait. This finding has been supported by the present study, as the *Ae. biuncialis* subpopulations A, B, D, and E showed significant differences in the heading time. The broad intraspecific diversity of the heading phenotypes could be introduced into bread wheat by means of chromosome-mediated gene transfer in order to diversify the maturation time and prolong the harvest period.

Besides the heading time, the stay-green trait, which is associated with foliar senescence characters, was also determined in the *Ae. biuncialis* accessions (Bachmann et al., 1994; Thomas et al., 1996). Delayed senescence allows the leaves to maintain photosynthetic activity during the active grain-filling period, thus ensuring the incorporation of assimilates to the grain (Thomas and Howarth, 2000; Spano et al., 2003; Kobata et al., 2015). The stay-green phenotype is associated with the ability of plants to maintain grain development longer, resulting in better yields, especially under water-limited conditions (Borrell et al., 2000; Christopher et al., 2008). In the present experiments, seven of the 86 *Ae. biuncialis* genotypes had SPAD values below the detection limit, due to premature leaf senescence. Eight accessions that exhibited the stay-green phenotype with early flowering were identified as potential gene sources for wheat breeding programs aimed to improve drought tolerance. Terminal drought during the grain-filling period can be avoided through accelerated reproduction, while the crop yield may be increased due to the preservation of the green leaf area.

## Conclusions

This paper confirmed that the DArTseq genotyping approach can be used efficiently to investigate the genetic diversity and population structure of *Ae. biuncialis*. The population structure defined using a combination of three clustering methods was in agreement with the geographic origin of the accessions. The heading trait diversity was also found to correlate largely with the genetic structure and geographic distribution. The wide-ranging genetic diversity of *Ae. biuncialis* may make it a potential genetic source for wheat improvement. The large intraspecific variation for heading time offers opportunities for the maximization of grain yield by shortening the plant life cycle, resulting in the termination of the grain-filling period before the drought season. The comparative analysis of genetic and phenological diversity patterns enables the selection of *Ae. biuncialis* genotypes adapted to a wide variety of ecological habitats, which could be used to breed new wheat cultivars able to cope with extreme climatic changes.

## Data Availability Statement

The SilicoDArT marker data of the *Ae. biuncialis* collection can be found in EBI https://www.ebi.ac.uk/biostudies/studies/S-BSST277.

## Author Contributions

LI participated in the analysis of the phenotypic data and wrote the manuscript. IMon performed the cluster analysis and made valuable comments on the manuscript. MM and PM maintained the germplasm and phenotyped it for the heading time. AF, ET, EG, AL-T, KS-P, and ÉS contributed to the maintenance of the plant material and the field experiments. ÉD carried out the phenotypic analysis of SPAD. TK evaluated the heading time of the genotypes. AK helped in GBS data analysis. IMol planned and managed the whole study, and supervised the manuscript preparation.

## Funding

This paper was financed by a Marie Curie Fellowship Grant (‘AEGILWHEAT’-H2020-MSCA-IF-2016-746253) under the H2020 framework program of the European Union and by the Hungarian National Research, Development and Innovation Office—NKFIH 116277, 112226, and 119387 and by the ERDF project „Plants as a tool for sustainable global development” (no. CZ.02.1.01/0.0/0.0/16_019/0000827).

## Conflict of Interest

The authors declare that the research was conducted in the absence of any commercial or financial relationships that could be construed as a potential conflict of interest.
