# Supplementary material for: Unlocking the Genetic Diversity and Population Structure of a Wild Gene Source of Wheat, Aegilops biuncialis Vis., and Its Relationship With the Heading Time
Source: Front Plant Sci. 2019 Nov 22;10:1531. doi: 10.3389/fpls.2019.01531 (PMC6882925; doi:10.3389/fpls.2019.01531)
Supplement: Supplementary file 2 [file DataSheet_2.docx]

**Supplementary material**

**Unlocking the genetic diversity and population structure of a wild gene source of wheat, *Ae. biuncialis* Vis., and its relationship with the heading time**

László Ivanizs^1a^, István Monostori^1a^, András Farkas^1^, Mária Megyeri^1^, Péter Mikó^1^, Edina Türkösi^1^, Eszter Gaál^1^, Andrea Lenykó-Thegze^1^, Kitti Szőke-Pázsi^1^, Éva Szakács^1^, Éva Darkó^1^, Tibor Kiss^1^, Andrzej Kilian^2^, István Molnár^1,3*^

^1^Agricultural Institute, Centre for Agricultural Research, Martonvásár, Hungary

^2^ Diversity Array Technologies, Canberra, ACT, Australia

^3^Institute of Experimental Botany, Center of the Region Haná for Biotechnological and Agricultural Research, Šlechtitelů 31, CZ-78371 Olomouc, Czech Republic

*** Correspondence:**István Molnár
molnar.istvan@agrar.mta.hu

^a^These authors are equal first authors

The following supplementary information is contained in these files:

**Supplementary Data S1.**

The file contains information (Identification number of the markers, trimmed sequences and the allelic composition on the 86 *Ae. biuncialis* accessions) for the row (unfiltered) set of 47777 SilicoDArT markers used in the present study.

Three tables as additional separate files-

**Table S1.** Availability of the *Ae. biuncialis* genotypes, including information about the place of origin.

**Table S2.** Genetic and phenological trait (heading time) diversification in the collection of *Aegilops biuncialis* genotypes.

**Table S3.** SPAD values measured in the *Ae. biuncialis* collection at two different times.

Ten figures are inserted in this file-

**Supplementary Figure S1.** Bar plot representing the probability of the assignation of *Ae. biuncialis* accessions to each subpopulation based on DArTseq markers for a hypothetical number of subpopulations (K) ranging from 2 to 7.

**Supplementary Figure S2.** Principal coordinate analysis plot generated from the genetic distances of *Ae. biuncialis* accessions, explaining 27.61% of the genetic variance.

**Supplementary Figure S3.** Principal coordinate analysis plot generated from the genetic distances of *Ae. biuncialis* accessions, explaining 16.96% of the genetic variance.

**Supplementary Figure S4.** Descriptive statistics for the heading time of the *Ae. biuncialis* collection.

**Supplementary Figure S5.** Distribution of monthly precipitation (**A**) and changes in daily mean temperature between January 1 and May 31 (**B**) in Martonvásár for three years.

**Supplementary Figure S6.** Scatter plot of principal components 2 and 3, explaining 13.28 % of the phenotypic variance in the heading time of the *Ae. biuncialis* collection.

**Supplementary Figure S7.** Scatter plot of principal components 1 and 3, explaining 89.43% of the phenotypic variance in the heading time of the *Ae. biuncialis* collection.

**Supplementary Figure S8.** SPAD values of the *Aegilops biuncialis* genotypes at the first measuring date (on may 12, 2018).

**Supplementary Figure S9.** SPAD values of the *Aegilops biuncialis* genotypes at the second measuring date (on may 31, 2018).

**Supplementary Figure S10.** Correlation between the heading time of the *Ae. biuncialis* accessions and the difference in the SPAD values.


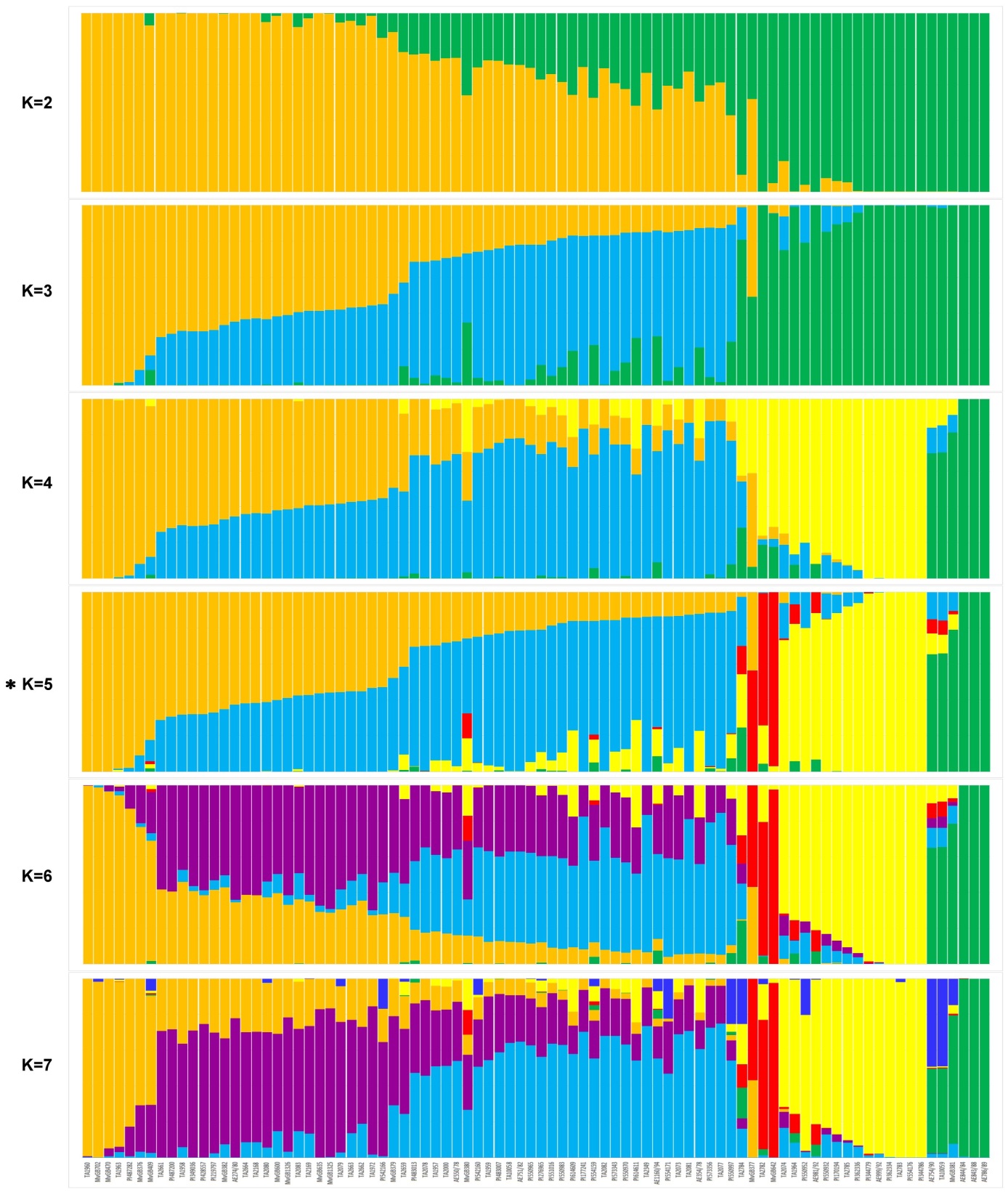


**Supplementary Figure S1.** Bar plot representing the probability of the assignation of *Ae. biuncialis* accessions to each subpopulation based on DArTseq markers for a hypothetical number of subpopulations (K) ranging from 2 to 7. The genetically most probable number of subpopulations is K = 5 (*). Each color represents a subpopulation and each column represents an accession. The length of the colored segments represents the estimated proportion of the subpopulations contributing to the genome of each accession.


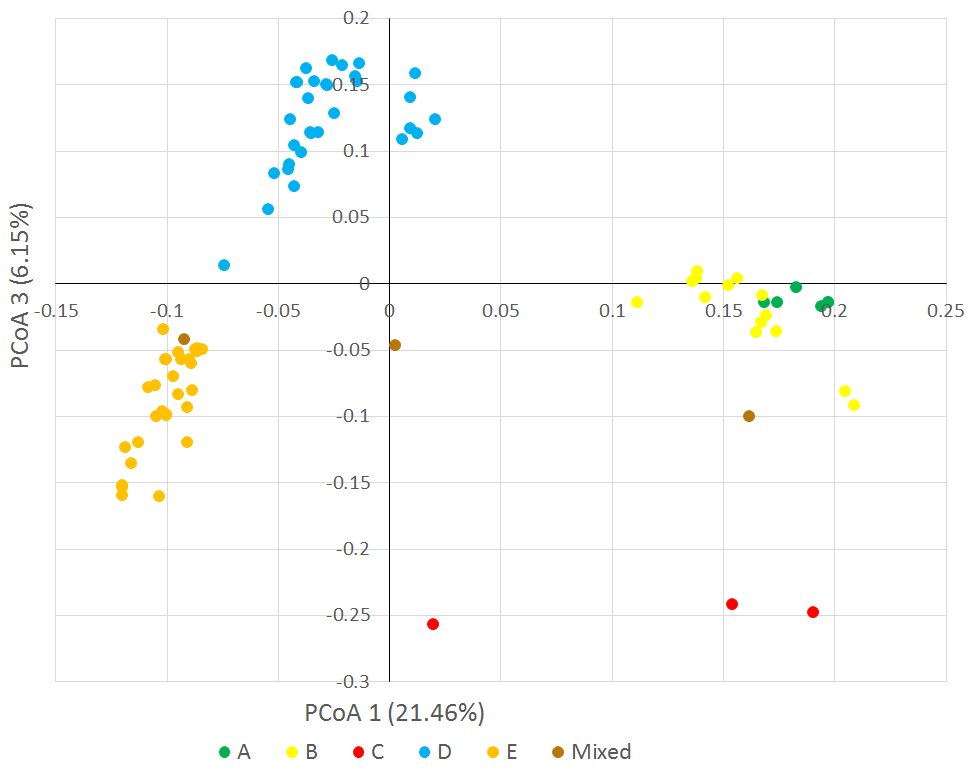


**Supplementary Figure S2.** Principal coordinate analysis plot generated from the genetic distances of *Ae. biuncialis* accessions, explaining 27.61% of the genetic variance. Color codes indicate different subpopulations (A-E) identified from STRUCTURE analysis based on >0.51% membership probability.


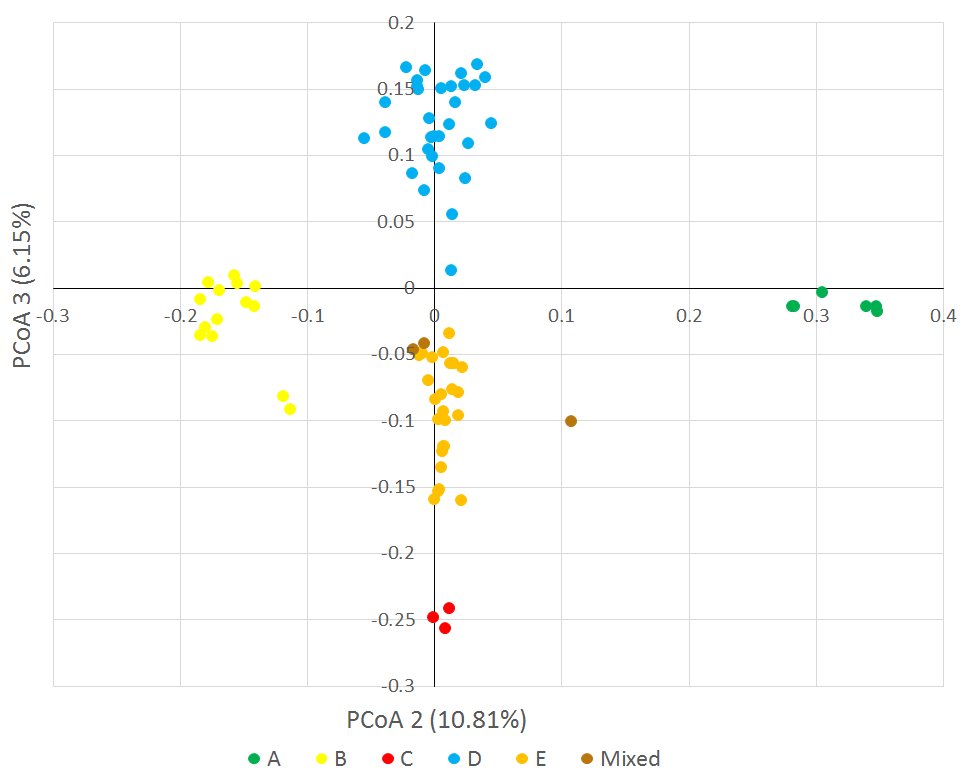


**Supplementary Figure S3.** Principal coordinate analysis plot generated from the genetic distances of *Ae. biuncialis* accessions explaining, 16.96% of the genetic variance. Color codes indicate different subpopulations (A-E) identified from STRUCTURE analysis based on >0.51% membership probability.


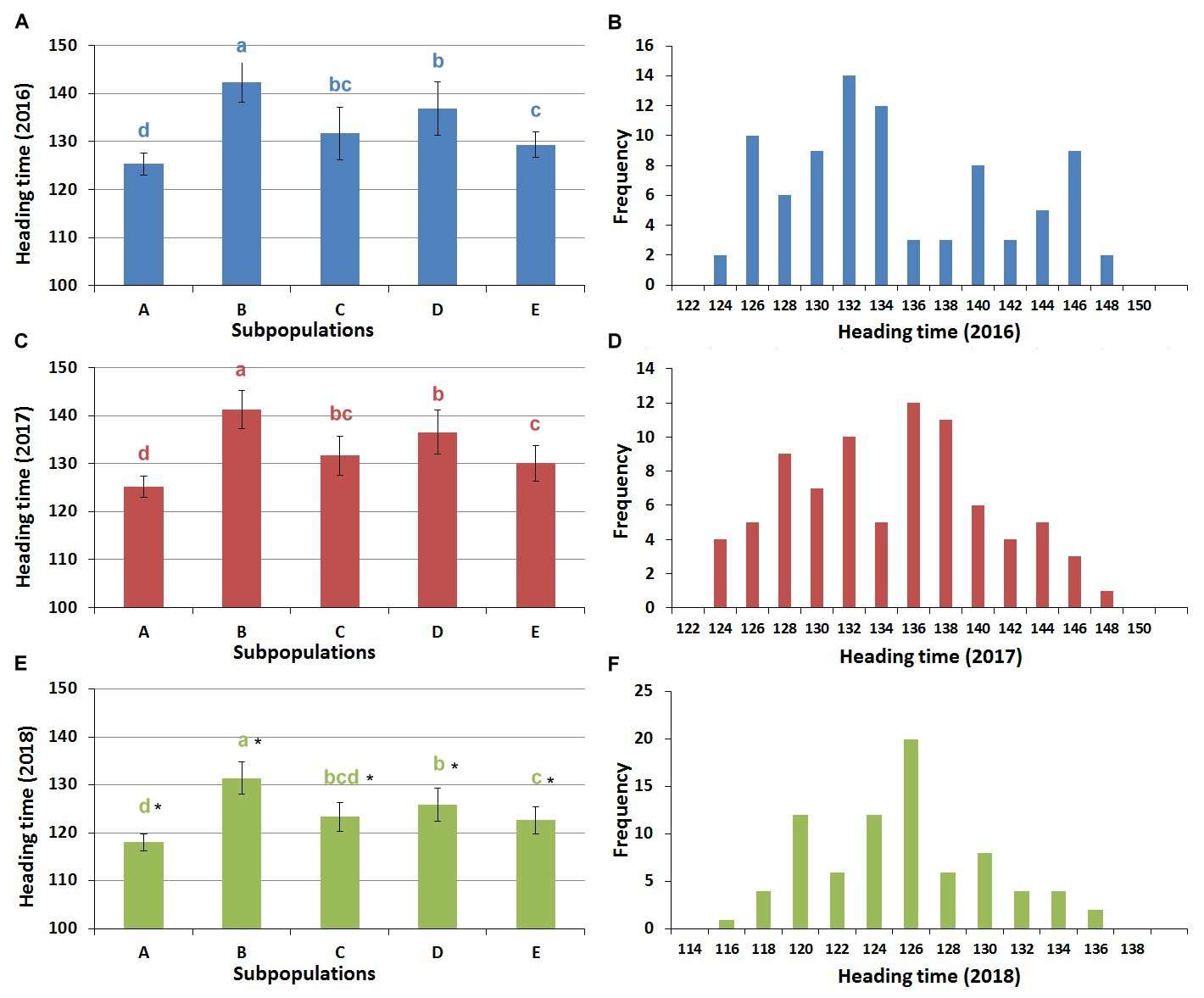


**Supplementary Figure S4.** Descriptive statistics for the heading time of the *Ae. biuncialis* collection. Distribution of the heading time (mean and SD) per subpopulation (**A**, **C** and **E**) and frequency distribution (**B**, **D** and **F**) in three consecutive years. Heading time was defined as the number of days required to reach the DEV59 developmental stage.

Different letters indicate significant differences between the subpopulations within the same year at P<0.05, using one-way ANOVA.

*indicates the heading time in 2018 differs significantly from the others within relevant subpopulation at the P<0.05 level.


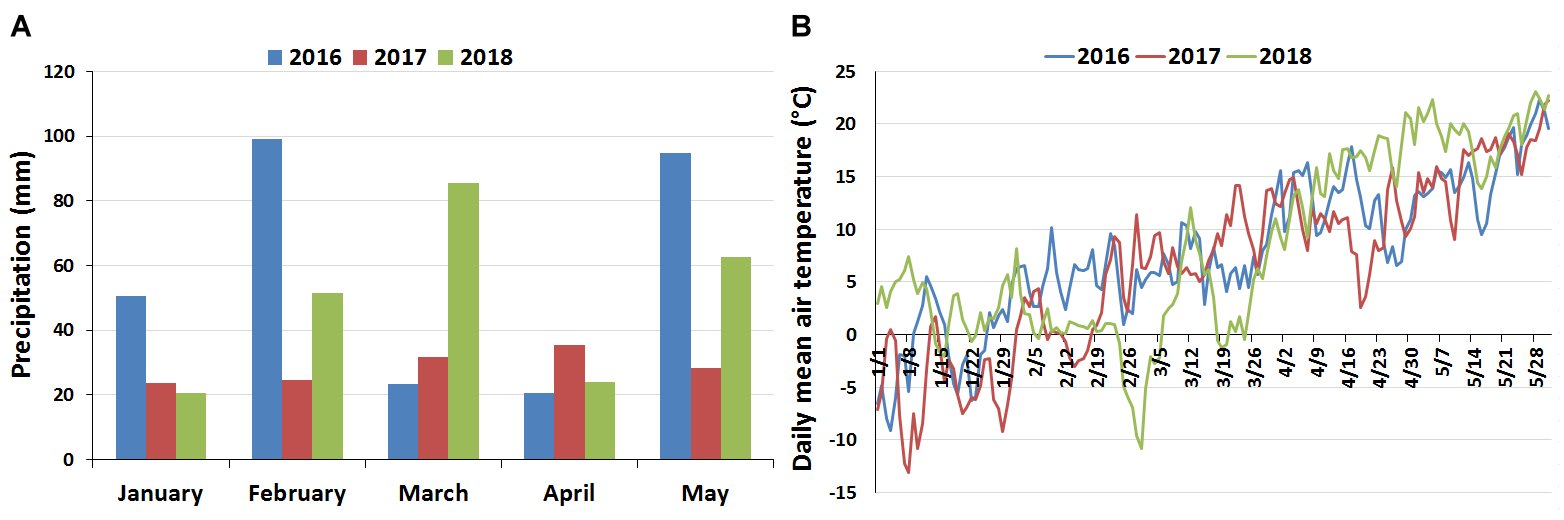


**Supplementary Figure S5.** Distribution of monthly precipitation (**A**) and changes in daily mean temperature between January 1 and May 31 (**B**) in Martonvásár for three years. The meteorological data (rainfall quantity and temperature) were measured at the local weather station.


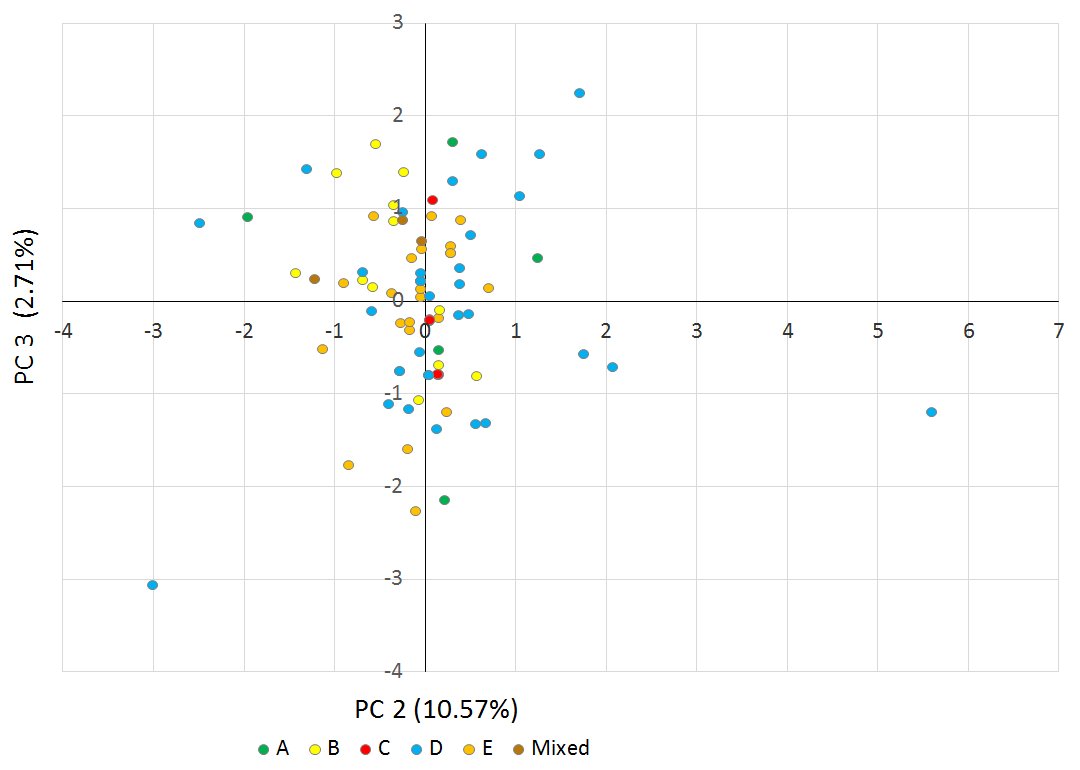


**Supplementary Figure S6.** Scatter plot of principal components 2 and 3, explaining 13.28 % of the phenotypic variance in the heading time of the *Ae. biuncialis* collection. Color codes indicate different subpopulations (A-E) identified from STRUCTURE analysis based on >0.51% membership probability.


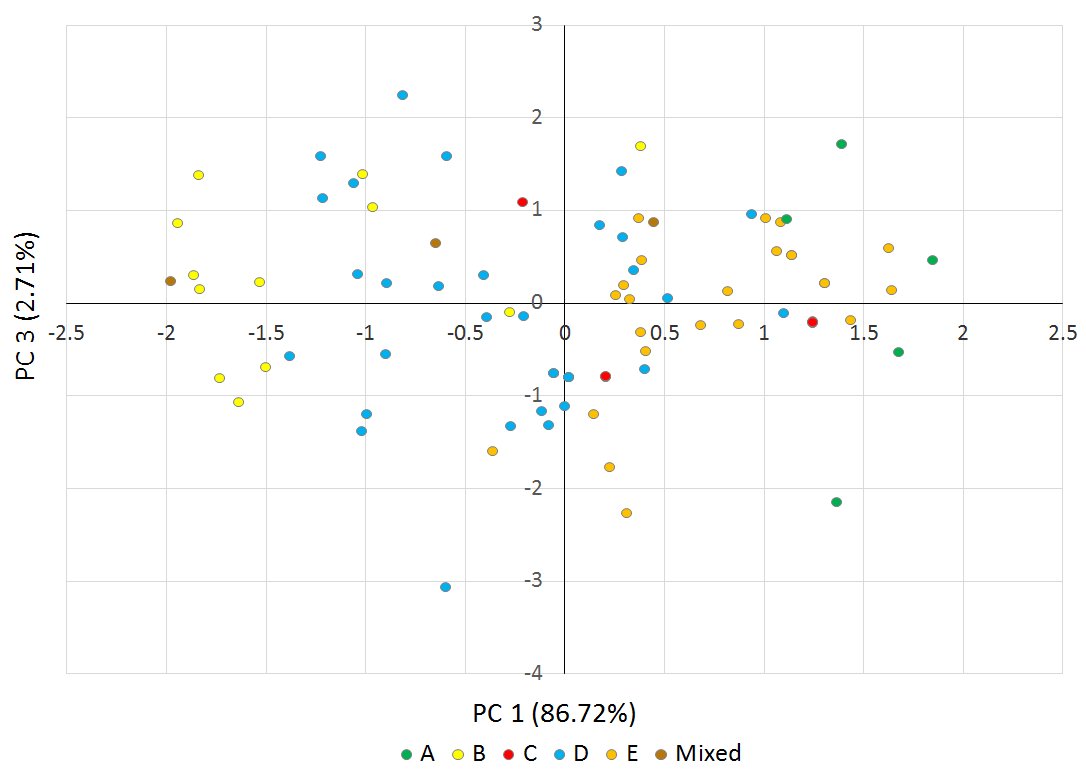


**Supplementary Figure S7.** Scatter plot of principal components 1 and 3, explaining 89.43% of the phenotypic variance in the heading time of the *Ae. biuncialis* collection. Color codes indicate different subpopulations (A-E) identified from STRUCTURE analysis based on >0.51% membership probability.


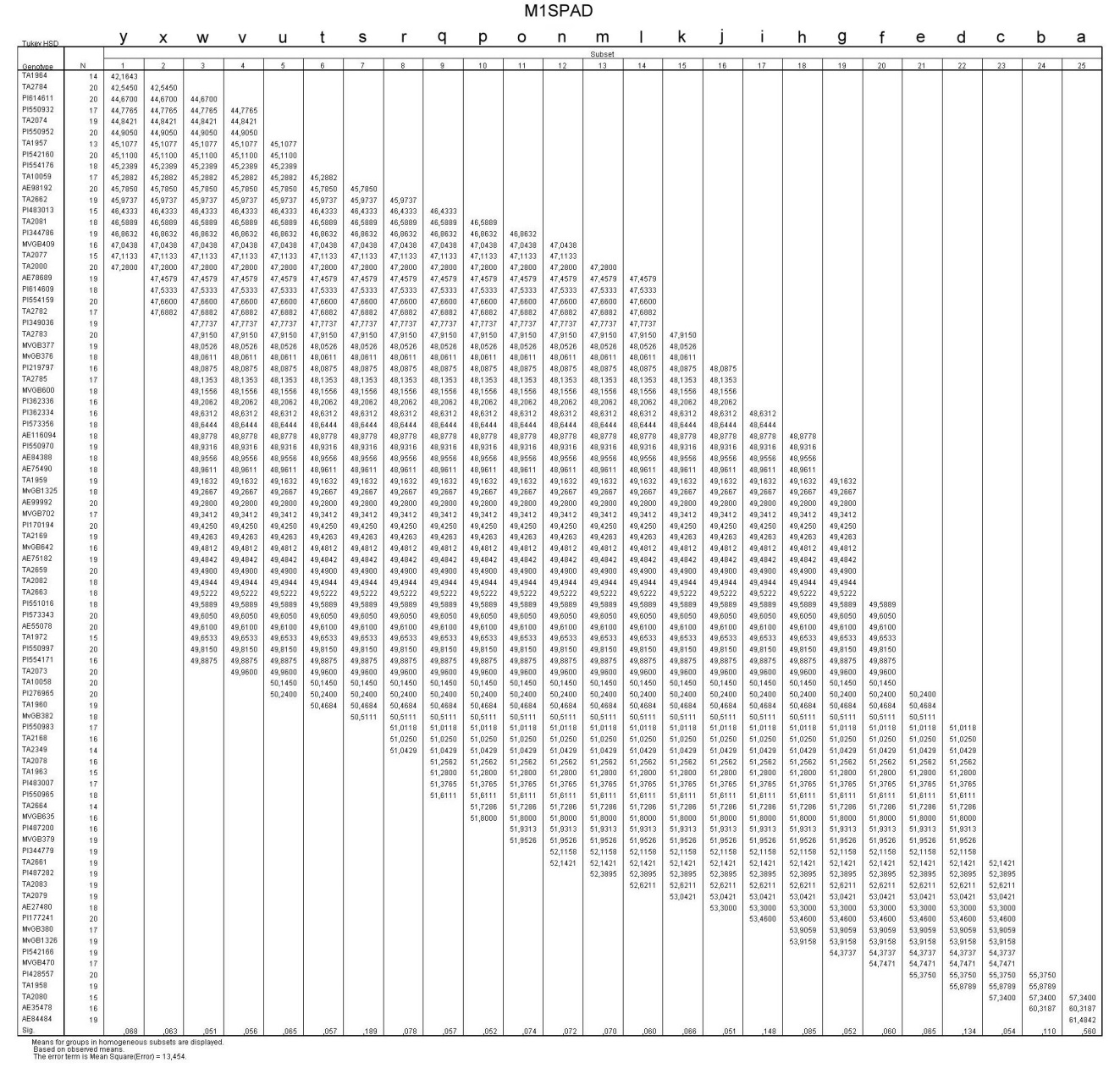


**Supplementary Figure S8.** SPAD values of the *Aegilops biuncialis* genotypes at the first measuring date (on may 12, 2018). The average SPAD value of genotypes is represented in the table. Tukey’s post hoc test was applied for statistacal analysis using SPSS 16.0 software. Differences were determined at the P<0.05 significance level.


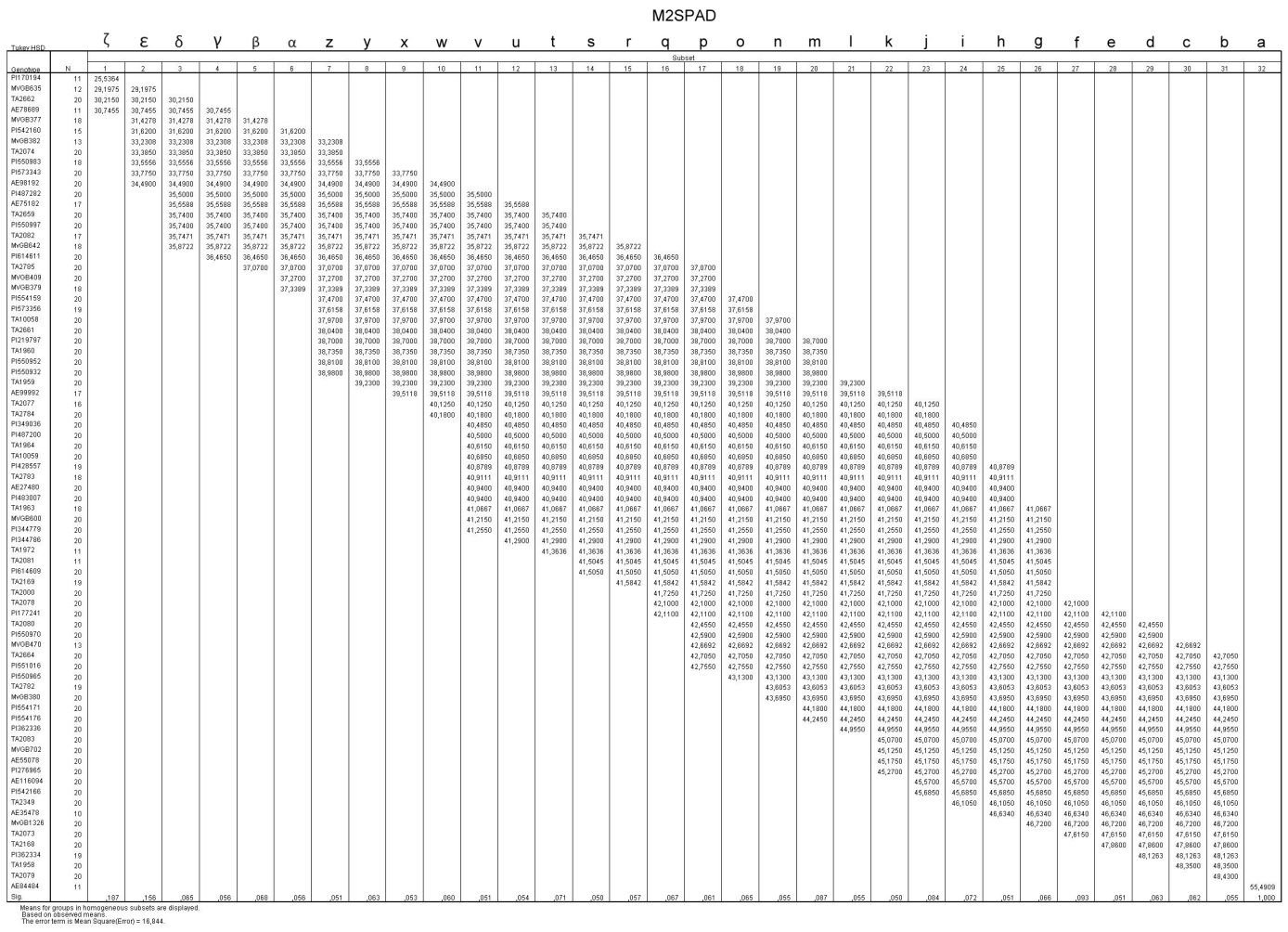


**Supplementary Figure S9.** SPAD values of the *Aegilops biuncialis* genotypes at the second measuring date (on may 31, 2018). The average SPAD value of genotypes is represented in the table. Tukey’s post hoc test was applied for statistacal analysis using SPSS 16.0 software. Differences were determined at the P<0.05 significance level.


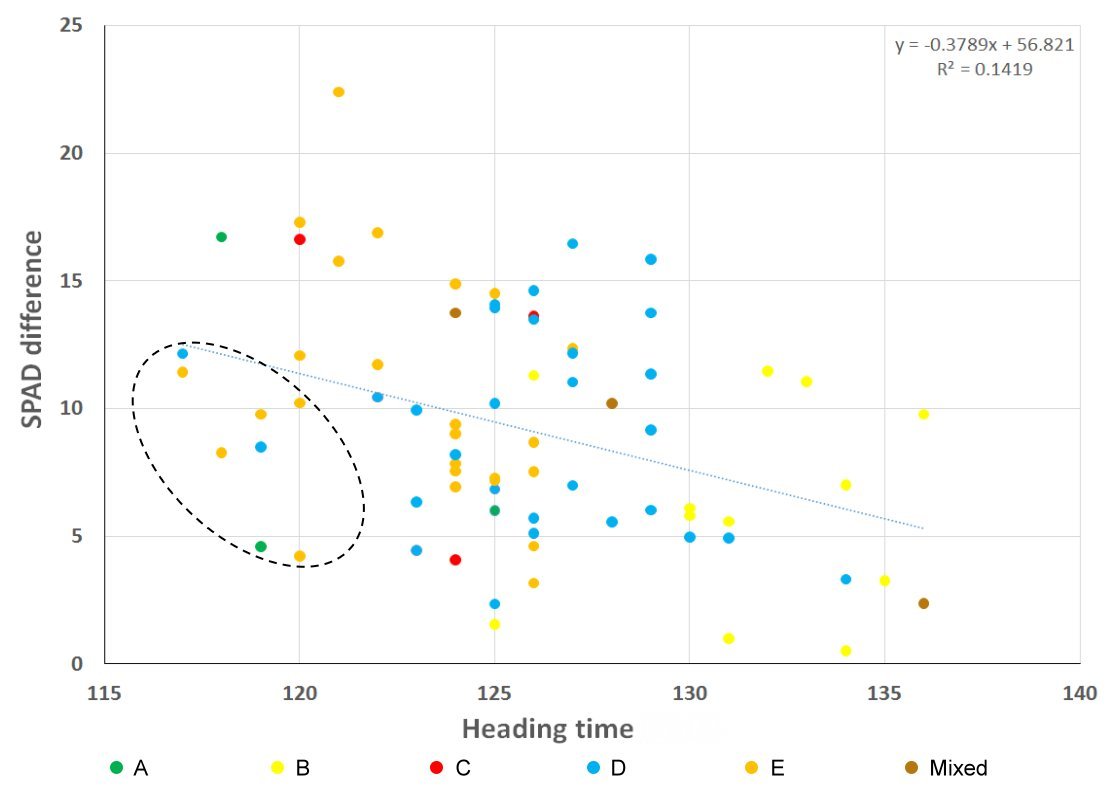


**Supplementary Figure S10.** Correlation between the heading time of the *Ae. biuncialis* accessions and the difference in the SPAD values. Different colored dots indicate *Ae. biuncialis* accessions belonging to different subpopulations (A-E) obtained by STRUCTURE analysis. The eight genotypes within the dotted-line circle showed stay-green and early heading time traits.
