# Supplementary material for: Unlocking the Genetic Diversity and Population Structure of a Wild Gene Source of Wheat, Aegilops biuncialis Vis., and Its Relationship With the Heading Time
Source: Front Plant Sci. 2019 Nov 22;10:1531. doi: 10.3389/fpls.2019.01531 (PMC6882925; doi:10.3389/fpls.2019.01531)
Supplement: Supplementary file 3 [file Table_1.docx]

**Table S1.** Availability of the *Ae. biuncialis* genotypes, including information about the place of origin.

| **Genotype^a^** | **Provenance (country, town)^b^** | **Elevation^c^** | **Permanent URL** |
| --- | --- | --- | --- |
| AE78689 | Libya, Alasaba | N/D | http://genbank.vurv.cz/ewdb/asp/ewdb_d2.asp?accn=155846 |
| AE84388 | Libya, Gharian | 550 m | http://genbank.vurv.cz/ewdb/asp/ewdb_d2.asp?accn=155845 |
| AE84484 | Libya, Ghadames | N/D | http://genbank.vurv.cz/ewdb/asp/ewdb_d2.asp?accn=155844 |
| MvGB381 | Unknown | N/D | N/D |
| TA10059 | Unknown | 30 m | https://purl.org/germplasm/id/25e35b83-fde9-40fe-982b-e412f30b6ef5 |
| AE75490 | Libya, Al Bayda | 450 m | http://genbank.vurv.cz/ewdb/asp/ewdb_d2.asp?accn=155850 |
| PI344786 | Serbia, Raska-Kopaonik | N/D | https://www.ars-grin.gov/npgs/pi_books/scans/pi177.pdf |
| PI554176 | Bosnia and Herzegovina, Mostar | 70 m | https://naldc.nal.usda.gov/download/43233/PDF |
| TA2783 | Bosnia and Herzegovina, Mostar | N/D | https://purl.org/germplasm/id/a65a6785-42fb-41c1-b112-9f21717556eb |
| PI362334 | Serbia, Kazinovici-Raska | 462 m | https://www.ars-grin.gov/npgs/pi_books/scans/pi179.pdf |
| AE99992 | Bulgaria | N/D | N/D |
| PI344779 | Macedonia, Tetovo | N/D | https://www.ars-grin.gov/npgs/pi_books/scans/pi177.pdf |
| PI362336 | Macedonia, Krivi Do | N/D | https://www.ars-grin.gov/npgs/pi_books/scans/pi179.pdf |
| TA2785 | Bosnia and Herzegovina, Mostar | N/D | https://purl.org/germplasm/id/071d17fa-d51b-49d2-81ae-b3aebb1b722c |
| PI170194 | Turkey, Kirklareli | N/D | https://www.ars-grin.gov/npgs/pi_books/scans/pi156.pdf |
| PI550932 | Greece, Larissa | 80 m | https://naldc.nal.usda.gov/download/43233/PDF |
| AE98192 | Greece, Lesbos | N/D | http://genbank.vurv.cz/ewdb/asp/ewdb_d2.asp?accn=155498 |
| PI550952 | Greece, Kilkis | 140 m | https://naldc.nal.usda.gov/download/43233/PDF |
| TA1964 | Turkey, Bergama | 65 m | https://purl.org/germplasm/id/8567f60f-4829-4e99-b178-477d2e1829c0 |
| TA2074 | Turkey, Cinarcik | N/D | https://purl.org/germplasm/id/2148512d-e2f6-4ef5-be98-e90b50a16c1c |
| MvGB642 | Syria, Slinfah | 1160 m | https://purl.org/germplasm/id/b2588cf9-e93e-45e1-88ea-1dd2f578e140 |
| TA2782 | Kosovo | N/D | https://purl.org/germplasm/id/5f065914-fc07-4760-9c52-62f1d41a0869 |
| MvGB377 | Jordan, Zai National Park | 1000 m | https://purl.org/germplasm/id/17fa2006-8892-413c-b8f3-ea052e48853f |
| PI550997 | Greece, Corinth | N/D | https://naldc.nal.usda.gov/download/43233/PDF |
| TA2077 | Turkey, Malatya-Sivas province border | 1500 m | https://purl.org/germplasm/id/4fad05ed-9590-4dbf-b798-d3e08a7d1afc |
| PI573356 | Turkey, Eskisehir | 770 m | https://www.ars-grin.gov/npgs/pi_books/scans/pi202pt4.pdf |
| AE35478 | Ukraine | N/D | http://genbank.vurv.cz/ewdb/asp/ewdb_d2.asp?accn=155855 |
| TA2081 | Turkey, Korkuteli | 1100 m | https://purl.org/germplasm/id/241a2221-c8ea-4e38-b7cd-77aa20ef38b1 |
| TA2073 | Turkey, Gemlik | 50 m | https://purl.org/germplasm/id/4852d0ca-2788-49b1-b841-0d4c6def9899 |
| PI554171 | Turkey, Izmir | 45 m | https://www.ars-grin.gov/npgs/pi_books/scans/pi200pt2.pdf |
| AE116094 | Greece, Ano Loussi | 1000 m | http://genbank.vurv.cz/ewdb/asp/ewdb_d2.asp?accn=155561 |
| TA2349 | Turkey, Kirsehir | 1020 m | https://purl.org/germplasm/id/fcf9aa91-76bb-4f70-8793-4798d0bdf9be |
| PI614611 | Crimea, Koktebel | 320 m | https://www.ars.usda.gov/ARSUserFiles/80420545/PIBooks/pibooks/plantinv209.pdf |
| PI550970 | Greece, Drossia | N/D | https://naldc.nal.usda.gov/download/43233/PDF |
| PI573343 | Turkey, Gemlik | 50 m | https://www.ars-grin.gov/npgs/pi_books/scans/pi202pt4.pdf |
| TA2082 | Turkey, Korkuteli | 900 m | https://purl.org/germplasm/id/bf4845d7-dddf-45d7-b3c4-47d7bec8ca35 |
| PI554159 | Turkey, Kusadasi | N/D | https://naldc.nal.usda.gov/download/43233/PDF |
| PI177241 | Turkey, Istambul | N/D | https://naldc.nal.usda.gov/download/39237/PDF |
| PI614609 | Crimea, Pryvitne | 40 m | https://www.ars.usda.gov/ARSUserFiles/80420545/PIBooks/pibooks/plantinv209.pdf |
| PI550983 | Greece, Argolis region | N/D | https://naldc.nal.usda.gov/download/43233/PDF |
| PI551016 | Greece, Amykles | N/D | https://naldc.nal.usda.gov/download/43233/PDF |
| PI276965 | Turkey | N/D | https://www.ars-grin.gov/npgs/pi_books/scans/pi169.pdf |
| PI550965 | Greece, Pikermi | 150 m | https://naldc.nal.usda.gov/download/43233/PDF |
| AE75182 | Turkey, Izmir | N/D | http://genbank.vurv.cz/ewdb/asp/ewdb_d2.asp?accn=155502 |
| TA10058 | Azerbaijan, Pirshaga | -3 m | https://purl.org/germplasm/id/f7b85247-e8ec-4e6a-8102-e47b3140999c |
| PI483007 | Cyprus, Nicosia | 160 m | https://www.ars-grin.gov/npgs/pi_books/scans/pi191.pdf |
| TA1959 | Turkey, Selendi | 490 m | https://purl.org/germplasm/id/5eca2758-a36d-438e-b952-a7d10704a86a |
| PI542160 | Turkey, Izmir | 60 m | https://www.ars-grin.gov/npgs/pi_books/scans/pi199pt2.pdf |
| AE55078 | France, Cevennes | N/D | http://genbank.vurv.cz/ewdb/asp/ewdb_d2.asp?accn=155967 |
| TA2000 | Turkey, Karacabey | 20 m | https://purl.org/germplasm/id/b43009d8-6c6f-4bc1-b5ba-ac79d1b26c8a |
| TA1957 | Turkey, Manisa | 490 m | https://purl.org/germplasm/id/217a7355-6aa2-4847-9943-ee88dcabc1f8 |
| TA2078 | Turkey, Elazig | 1000 m | https://purl.org/germplasm/id/5163bca6-cf0e-4a76-8cc5-b822b2a3217b |
| PI483013 | Cyprus, Larnaca | 200 m | https://www.ars-grin.gov/npgs/pi_books/scans/pi191.pdf |
| MvGB379 | Syria | N/D | N/D |
| PI542166 | Turkey, Gaziantep | 880 m | https://www.ars-grin.gov/npgs/pi_books/scans/pi199pt2.pdf |
| TA1972 | Unknown | N/D | https://purl.org/germplasm/id/eb0210f2-9fd4-43c7-b4a4-d37d07786fba |
| TA2662 | Syria, Sarmada | 550 m | https://purl.org/germplasm/id/d1c12d0d-bc51-437c-87c4-8ff44721dcf6 |
| TA2663 | Syria, Harem | 400 m | https://purl.org/germplasm/id/a5dc51e8-3729-4dda-a380-a25eef8a9a6c |
| TA2079 | Turkey, Gaziantep | 800 m | https://purl.org/germplasm/id/4137a26a-060e-412b-ae3e-68851cec552d |
| MvGB1325 | Azerbaijan, Baku-Schamakhi | 500 m | N/D |
| MvGB635 | Azerbaijan, Baku | N/D | N/D |
| TA2169 | Syria | N/D | https://purl.org/germplasm/id/0a2a51b9-df54-4e64-b2db-12c30b654d8b |
| TA2083 | Turkey, Hassa-Kilis | 500 m | https://purl.org/germplasm/id/cfc5a4c5-c646-466f-86f7-abc3d3bb7366 |
| MvGB1326 | Azerbaijan, Girdmanchay river | 319 m | N/D |
| MvGB600 | Unknown | N/D | N/D |
| TA2080 | Turkey, Araban | 820 m | https://purl.org/germplasm/id/af547889-9ee7-4730-b674-32dfd47eb917 |
| TA2168 | Syria | N/D | https://purl.org/germplasm/id/d94c55f0-1881-414f-b43d-2fdc301587ff |
| TA2664 | Syria, Harem | 400 m | https://purl.org/germplasm/id/2f298cc2-e465-447c-a3fa-142cf5aea72d |
| AE27480 | Azerbaijan | N/D | http://genbank.vurv.cz/ewdb/asp/ewdb_d2.asp?accn=155858 |
| MvGB382 | Syria | N/D | N/D |
| PI219797 | Iraq, Erbil | 915 m | https://www.ars-grin.gov/npgs/pi_books/scans/pi162.pdf |
| PI428557 | Azerbaijan | N/D | https://www.ars-grin.gov/npgs/pi_books/scans/pi186.pdf |
| PI349036 | Azerbaijan | N/D | https://naldc.nal.usda.gov/download/39205/PDF |
| TA1958 | Turkey, Kilis | 500 m | https://purl.org/germplasm/id/afa096dc-ba24-469a-8684-9d50f67e1c1e |
| PI487200 | Syria, Aleppo | 350 m | https://www.ars-grin.gov/npgs/pi_books/scans/pi192.pdf |
| TA2661 | Syria | N/D | https://purl.org/germplasm/id/ffcdb0a0-7362-42b1-9d1c-d30287ece0ca |
| MvGB409 | Jordan, Kufr Khall | N/D | N/D |
| MvGB376 | Jordan, El Jubeiha | 875 m | https://purl.org/germplasm/id/8a0db7c4-bf6e-4a92-b511-c6c424d3dce1 |
| PI487282 | Jordan, Irbid | N/D | https://www.ars-grin.gov/npgs/pi_books/scans/pi192.pdf |
| TA1963 | Unknown | N/D | https://purl.org/germplasm/id/13d03e60-f29b-4b54-98a3-ca9d4362b246 |
| MvGB470 | Jordan, Anjara | 740 m | https://purl.org/germplasm/id/d3629e29-9004-4814-aadf-28a104c5a833 |
| MvGB702 | Jordan, Al-Salt | 750 m | https://purl.org/germplasm/id/8bbfa06a-f72c-4b20-b631-71713c9d1be6 |
| TA1960 | Israel, Jerusalem | 756 m | https://purl.org/germplasm/id/b9d9cf2c-29bb-4d3c-b00c-fdf2ce418548 |
| MvGB380 | Unknown | N/D | N/D |
| TA2784 | Bosnia and Herzegovina, Mostar | N/D | https://purl.org/germplasm/id/ee9180fb-b815-4c63-8721-ef2a4d849232 |
| TA2659 | Syria, Zabadani | 1180 m | https://purl.org/germplasm/id/9be98a00-f626-4fa9-a4e4-75a42f3bbc2e |

**^a^** ID name of donor accessions

**^bc^** Geographic origin and metres above sea level from Plant Inventory Books: USDA ARS, European Wheat Database and Genesys Accession browser. (<https://www.ars.usda.gov/northeast-area/beltsville-md-barc/beltsville-agricultural-research-center/national-germplasm-resources-laboratory/docs/plant-inventory-books/>, <https://www.genesys-pgr.org/explore>, <http://genbank.vurv.cz/ewdb/>)

MvGB, accessions from the Martonvásár (ATK) gene bank; TA, accessions from the Wheat Genetics Resource Center; PI, accessions from USDA ARS and AE, accessions from the Institute of Plant Genetics and Crop Plant Research.
