# Supplementary material for: Unlocking the Genetic Diversity and Population Structure of a Wild Gene Source of Wheat, Aegilops biuncialis Vis., and Its Relationship With the Heading Time
Source: Front Plant Sci. 2019 Nov 22;10:1531. doi: 10.3389/fpls.2019.01531 (PMC6882925; doi:10.3389/fpls.2019.01531)
Supplement: Supplementary file 4 [file Table_2.docx]

**Table S2.** Genetic and phenological trait (heading time) diversification in the collection of *Aegilops biuncialis* genotypes.

| **Genotype^a^** | **Subpopulation^b^** | **Heading time 2016^c^** | **Heading time 2017^c^** | **Heading time 2018^c^** |
| --- | --- | --- | --- | --- |
| AE78689 | A | 123 | 125 | 118 |
| AE84388 | A | 125 | 124 | 115 |
| AE84484 | A | 125 | 124 | 125 |
| MvGB381 | A | 128 | N/D | N/D |
| TA10059 | A | 123 | 129 | 119 |
| AE75490 | A | 128 | 124 | 119 |
| PI344786 | B | 145 | 146 | 131 |
| PI554176 | B | 143 | 144 | 131 |
| TA2783 | B | 145 | 144 | 134 |
| PI362334 | B | 147 | 144 | 134 |
| AE99992 | B | 144 | 143 | 136 |
| PI344779 | B | N/D | N/D | N/D |
| PI362336 | B | 146 | 142 | 135 |
| TA2785 | B | 143 | 142 | 133 |
| PI170194 | B | N/D | N/D | N/D |
| PI550932 | B | 141 | 138 | 130 |
| AE98192 | B | 136 | 136 | 126 |
| PI550952 | B | 142 | 138 | 130 |
| TA1964 | B | 133 | 133 | 125 |
| TA2074 | B | 143 | 145 | 132 |
| MvGB642 | C | 137 | 134 | 126 |
| TA2782 | C | 132 | 134 | 124 |
| MvGB377 | C | 126 | 127 | 120 |
| PI550997 | D | 145 | 138 | 125 |
| TA2077 | D | 137 | 136 | 127 |
| PI573356 | D | 145 | 145 | 127 |
| AE35478 | D | 148 | 148 | 117 |
| TA2081 | D | 133 | 135 | 126 |
| TA2073 | D | 142 | 137 | 125 |
| PI554171 | D | 132 | 135 | 126 |
| AE116094 | D | 129 | 138 | 134 |
| TA2349 | D | 140 | 139 | 131 |
| PI614611 | D | 134 | 137 | 124 |
| PI550970 | D | 133 | 132 | 123 |
| PI573343 | D | 139 | 142 | 129 |
| TA2082 | D | 139 | 140 | 129 |
| PI554159 | D | 135 | 138 | 125 |
| PI177241 | D | 143 | 139 | 129 |
| PI614609 | D | 140 | 139 | 129 |
| PI550983 | D | 139 | 138 | 127 |
| PI551016 | D | 136 | 136 | 125 |
| PI276965 | D | 140 | 139 | 130 |
| PI550965 | D | 134 | 135 | 119 |
| AE75182 | D | 133 | 132 | 125 |
| TA10058 | D | 132 | 129 | 127 |
| PI483007 | D | 129 | 127 | 122 |
| TA1959 | D | 134 | 132 | 123 |
| PI542160 | D | 137 | 137 | 126 |
| AE55078 | D | 131 | 131 | 123 |
| TA2000 | D | 145 | 141 | 128 |
| TA1957 | D | 133 | 135 | 125 |
| TA2078 | D | 145 | 140 | 129 |
| PI483013 | D | 126 | 127 | 122 |
| MvGB379 | D | 133 | 136 | 126 |
| PI542166 | E | 129 | 134 | 126 |
| TA1972 | E | 125 | 124 | 118 |
| TA2662 | E | 128 | 127 | 121 |
| TA2663 | E | 129 | 130 | 123 |
| TA2079 | E | 131 | 131 | 126 |
| MvGB1325 | E | 129 | 127 | 120 |
| MvGB635 | E | 129 | 127 | 121 |
| TA2169 | E | 131 | 132 | 124 |
| TA2083 | E | 132 | 134 | 124 |
| MvGB1326 | E | 132 | 130 | 125 |
| MvGB600 | E | 132 | 132 | 124 |
| TA2080 | E | 132 | 135 | 124 |
| TA2168 | E | 129 | 131 | 126 |
| TA2664 | E | 132 | 131 | 124 |
| AE27480 | E | 134 | 138 | 127 |
| MvGB382 | E | 126 | 127 | 120 |
| PI219797 | E | 129 | 135 | 124 |
| PI428557 | E | 132 | 132 | 125 |
| PI349036 | E | 133 | 135 | 125 |
| TA1958 | E | 133 | 135 | 126 |
| PI487200 | E | 125 | 125 | 117 |
| TA2661 | E | N/D | N/D | N/D |
| MvGB409 | E | 125 | 126 | 119 |
| MvGB376 | E | 126 | 126 | 120 |
| PI487282 | E | 128 | 129 | 122 |
| TA1963 | E | 128 | 127 | 120 |
| MvGB470 | E | 128 | 127 | 120 |
| MvGB702 | E | 126 | 126 | 120 |
| TA1960 | E | 129 | 129 | 122 |
| MvGB380 | Mixed | 139 | 137 | 128 |
| TA2784 | Mixed | 145 | 144 | 136 |
| TA2659 | Mixed | 132 | 130 | 124 |

**^a^** ID name of donor accessions

**^b^** Subpopulations of the *Ae. biuncialis* collection based on cluster analysis

**^c^** Heading time was determined as number of days elapsing from January 1 to ear emergence (DEV59 developmental stage) in three growing seasons.
