# Supplementary material for: Unlocking the Genetic Diversity and Population Structure of a Wild Gene Source of Wheat, Aegilops biuncialis Vis., and Its Relationship With the Heading Time
Source: Front Plant Sci. 2019 Nov 22;10:1531. doi: 10.3389/fpls.2019.01531 (PMC6882925; doi:10.3389/fpls.2019.01531)
Supplement: Supplementary file 5 [file Table_3.docx]

**Table S3.** SPAD values measured in the *Ae. biuncialis* collection at two different times.

| **Genotypes^a^** | **SPAD measurement 1^b^** | **SPAD measurement 2^c^** | **SPAD difference^d^** |
| --- | --- | --- | --- |
| AE78689 | 47.46±4.46 | 30.74±5.44 | 16.72 |
| AE84388**^1^** | 48.96±3.42 | N/D | N/D |
| AE84484 | 61.48±3.76 | 55.49±4.14 | 5.99 |
| MVGB381 | N/D | N/D | N/D |
| TA10059* | 45.29±3.27 | 40.69±3.39 | 4.60 |
| AE75490**^1^** | 48.96±3.55 | N/D | N/D |
| PI344786 | 46.86±4.07 | 41.29±3.17 | 5.57 |
| PI554176 | 45.24±2.81 | 44.25±4.44 | 0.99 |
| TA2783 | 47.92±3.92 | 40.91±3.22 | 7.00 |
| PI362334 | 48.63±2.57 | 48.13±3.34 | 0.50 |
| AE99992 | 49.28±3.29 | 39.51±5.17 | 9.77 |
| PI344779 | 52.12±4.45 | 41.26±3.81 | 10.86 |
| PI362336 | 48.21±3.11 | 44.96±3.50 | 3.25 |
| TA2785 | 48.14±3.01 | 37.07±6.42 | 11.07 |
| PI170194 | 49.43±3.37 | 25.54±3.73 | 23.89 |
| PI550932 | 44.78±3.49 | 38.98±3.56 | 5.80 |
| AE98192 | 45.79±3.83 | 34.49±3.10 | 11.30 |
| PI550952 | 44.91±3.12 | 38.81±3.10 | 6.09 |
| TA1964 | 42.16±2.79 | 40.62±4.50 | 1.55 |
| TA2074 | 44.84±2.44 | 33.39±3.92 | 11.46 |
| MvGB642 | 49.48±3.59 | 35.87±4.79 | 13.61 |
| TA2782 | 47.69±3.63 | 43.61±3.45 | 4.08 |
| MVGB377 | 48.05±3.06 | 31.43±4.81 | 16.62 |
| PI550997 | 49.82±3.08 | 35.74±4.92 | 14.08 |
| TA2077 | 47.11±4.04 | 40.13±4.43 | 6.99 |
| PI573356 | 48.64±3.81 | 37.62±5.26 | 11.03 |
| AE35478* | 60.32±5.35 | 48.18±10.48 | 12.14 |
| TA2081 | 46.59±3.47 | 41.49±2.24 | 5.10 |
| TA2073 | 49.96±4.40 | 47.62±2.96 | 2.35 |
| PI554171 | 49.89±3.82 | 44.18±3.26 | 5.71 |
| AE116094 | 48.88±2.98 | 45.57±3.23 | 3.31 |
| TA2349 | 51.04±3.86 | 46.11±5.04 | 4.94 |
| PI614611 | 44.67±3.55 | 36.47±3.02 | 8.21 |
| PI550970 | 48.93±5.20 | 42.59±4.54 | 6.34 |
| PI573343 | 49.61±4.57 | 33.78±3.72 | 15.83 |
| TA2082 | 49.49±4.81 | 35.75±5.10 | 13.75 |
| PI554159 | 47.66±3.14 | 37.47±3.16 | 10.19 |
| PI177241 | 53.46±3.42 | 42.11±4.14 | 11.35 |
| PI614609 | 47.53±3.40 | 41.51±3.34 | 6.03 |
| PI550983 | 50.01±3.79 | 33.56±6.08 | 16.45 |
| PI551016 | 49.59±4.73 | 42.76±3.82 | 6.83 |
| PI276965 | 50.24±3.59 | 45.27±4.32 | 4.97 |
| PI550965* | 51.61±5.80 | 43.13±3.31 | 8.48 |
| AE75182 | 49.48±3.23 | 35.56±4.85 | 13.93 |
| TA10058 | 50.15±4.62 | 37.97±3.83 | 12.18 |
| PI483007 | 51.38±4.47 | 40.94±5.02 | 10.44 |
| TA1959 | 49.16±3.90 | 39.23±3.51 | 9.93 |
| PI542160 | 45.11±2.85 | 31.62±3.55 | 13.49 |
| AE55078 | 49.61±3.23 | 45.18±3.88 | 4.44 |
| TA2000 | 47.28±3.45 | 41.73±2.63 | 5.56 |
| TA1957**^1^** | 45.11±2.75 | N/D | N/D |
| TA2078 | 51.26±4.30 | 42.10±3.41 | 9.16 |
| PI483013**^1^** | 46.43±3.81 | N/D | N/D |
| MVGB379 | 51.95±3.09 | 37.34±4.77 | 14.61 |
| PI542166 | 54.36±2.60 | 45.69±4.57 | 8.68 |
| TA1972* | 49.65±3.17 | 41.39±3.57 | 8.26 |
| TA2662 | 45.97±3.09 | 30.22±5.04 | 15.76 |
| TA2663**^1^** | 49.52±3.35 | N/D | N/D |
| TA2079 | 53.04±2.86 | 48.43±3.01 | 4.61 |
| MvGB1325**^1^** | 49.27±2.36 | N/D | N/D |
| MVGB635 | 51.80±3.96 | 29.40±4.15 | 22.40 |
| TA2169 | 49.43±2.83 | 41.58±5.7 | 7.84 |
| TA2083 | 52.62±4.72 | 45.07±3.18 | 7.55 |
| MvGB1326 | 53.92±3.36 | 46.72±3.84 | 7.20 |
| MVGB600 | 48.16±4.30 | 41.22±3.85 | 6.94 |
| TA2080 | 57.34±3.90 | 42.46±4.50 | 14.89 |
| TA2168 | 51.03±3.01 | 47.86±3.58 | 3.17 |
| TA2664 | 51.73±2.68 | 42.71±3.43 | 9.02 |
| AE27480 | 53.30±3.41 | 40.94±4.34 | 12.36 |
| MvGB382 | 50.51±2.76 | 33.23±5.39 | 17.28 |
| PI219797 | 48.09±3.97 | 38.70±4.51 | 9.39 |
| PI428557 | 55.38±4.28 | 40.88±4.23 | 14.50 |
| PI349036 | 47.77±4.18 | 40.49±3.56 | 7.29 |
| TA1958 | 55.88±3.84 | 48.35±3.36 | 7.53 |
| PI487200* | 51.93±3.58 | 40.50±4.93 | 11.43 |
| TA2661 | 52.14±3.52 | 38.04±4.04 | 14.10 |
| MVGB409* | 47.04±2.60 | 37.27±5.06 | 9.77 |
| MvGB376**^1^** | 48.06±3.75 | N/D | N/D |
| PI487282 | 52.39±3.31 | 35.50±3.15 | 16.89 |
| TA1963* | 51.28±5.17 | 41.07±4.01 | 10.21 |
| MVGB470 | 54.75±2.78 | 42.67±3.34 | 12.08 |
| MVGB702* | 49.34±2.91 | 45.13±3.57 | 4.22 |
| TA1960 | 50.47±2.73 | 38.74±3.73 | 11.73 |
| MvGB380 | 53.91±4.10 | 43.70±4.71 | 10.21 |
| TA2784 | 42.55±3.66 | 40.18±2.97 | 2.36 |
| TA2659 | 49.49±3.56 | 35.74±4.52 | 13.75 |

Data represent mean ± standard deviation of at least 10 plants per genotype. The results of statistical analysis are presented in **Supplementary Figure S8 and S9**.

**^a^** ID name of donor accessions

**^b^** First measurement of SPAD values on May 12, 2018

**^c^** Second measurement of SPAD values on May 31, 2018

**^d^** Difference between the SPAD values at the two measurement times

**^1^** *Ae. biuncialis* accessions with non-detectable SPAD value at the second measurement time

* *Ae. biuncialis* accessions with stay-green trait and early heading time
